# DNA hairpins destabilize duplexes primarily by promoting melting rather than by inhibiting hybridization

**DOI:** 10.1093/nar/gkv582

**Published:** 2015-06-08

**Authors:** John S. Schreck, Thomas E. Ouldridge, Flavio Romano, Petr Šulc, Liam P. Shaw, Ard A. Louis, Jonathan P.K. Doye

**Affiliations:** 1 Physical and Theoretical Chemistry Laboratory, Department of Chemistry, University of Oxford, South Parks Road, Oxford OX1 3QZ, UK; 2 Rudolf Peierls Centre for Theoretical Physics, University of Oxford, 1 Keble Road, Oxford OX1 3NP, UK; 3 Department of Mathematics, Imperial College, 180 Queen's Gate, London, SW7 2AZ, UK; 4 Center for Studies in Physics and Biology, The Rockefeller University, 1230 York Avenue, New York, NY 10065, USA

## Abstract

The effect of secondary structure on DNA duplex formation is poorly understood. Using oxDNA, a nucleotide level coarse-grained model of DNA, we study how hairpins influence the rate and reaction pathways of DNA hybridzation. We compare to experimental systems studied by Gao *et al*. (1) and find that 3-base pair hairpins reduce the hybridization rate by a factor of 2, and 4-base pair hairpins by a factor of 10, compared to DNA with limited secondary structure, which is in good agreement with experiments. By contrast, melting rates are accelerated by factors of ∼100 and ∼2000. This surprisingly large speed-up occurs because hairpins form during the melting process, and significantly lower the free energy barrier for dissociation. These results should assist experimentalists in designing sequences to be used in DNA nanotechnology, by putting limits on the suppression of hybridization reaction rates through the use of hairpins and offering the possibility of deliberately increasing dissociation rates by incorporating hairpins into single strands.

## INTRODUCTION

The field of DNA nanotechnology has grown enormously since Seeman's original work in the early 1980s, which suggested that the specificity of DNA hybridization could be harnessed to permit the design of artificial structures (2). Large-scale structures can now be designed to self-assemble with nanoscale precision (3–6). DNA based switches and motors have been demonstrated (7–11), and decision-making constructs have been shown to interact with biological systems (12). Other work has explored the potential for DNA-based computation (13–15). The fundamental ingredient in the self-assembly of DNA nanostructures and in the operation of DNA nanomachines is the hybridization of single-stranded DNA to form duplexes.

Besides the canonical double-helical duplex, DNA hairpins, in which a self-complementary strand loops around to bind to itself, are perhaps the simplest structure that DNA can form. In nanotechnological applications, hairpins have the potential to be both advantageous and deleterious. For example, metastable hairpins may impede hybridization by blocking binding sites, thereby providing an additional barrier to hybridization (1). This can be a nuisance for DNA nanostructures that are assembled at low temperature, especially those structures that are composed of longer strands, since such single strands are likely to possess intra-strand base pairs that will inhibit assembly (16). On the other hand, hairpins can also be used constructively to control some features of reaction pathways. For example, metastable hairpins can be a source of fuel for autonomous DNA machines (8,11,17,18), and can be used to suppress undesirable leak reactions (19).

Despite the importance of hairpins to DNA nanotechnology, a systematic understanding of their influence on the hybridization transition remains elusive. Recently, however, an important step in this direction was made by Gao *et al*. who measured the rate of hybridization at 20°C and high salt for several systems of complementary strands, where the strands contained either no stable hairpins, or stable 3- or 4-base pair hairpins (1). The hybridization rates of the 3-base pair hairpins were reduced by a factor of approximately 2, and those for the 4-base pair hairpins by an order of magnitude. While these are not negligible effects, they are smaller than might naïvely be expected. Assuming a second-order duplex formation transition, the hybridization (*k*_+_) and melting (*k*_−_) rate constants are necessarily related by the free-energy change of duplex formation Δ*G*^0^ with respect to the single-stranded state through

(1) \begin{equation*} \frac{k_+}{k_-} = \frac{\exp (-\Delta G^0/RT)}{c^0}, \end{equation*}

where *c*^0^ = 1 M. Gao *et al*. found that both the 3- and 4-base pair hairpins were very stable at 20°C (1), suggesting a significant change in Δ*G*^0^ relative to the hairpin-free system (particularly in the 4-base pair case). However, the measured reduction in the hybridization rate *k*_+_ is much less than the reduction in exp ( − Δ*G*^0^/*RT*) from Eq. (1). Although not noted by Gao *et al*., the experiments therefore strongly suggest, in fact, that hairpins lead to a large *increase* in the melting rate *k*_−_. Since the stable hairpins must first open in order for strands to form a duplex, it is not surprising that they slow down hybridization rates, as has been noted elsewhere (8,18,20–22). The influence of hairpins on dissociation rates has received little attention, perhaps because it seems natural to assume that hairpins only re-form after the melting process is complete. The results of Gao *et al*., however, point to a much stronger influence of hairpins on dissociation (1).

To explore the general physical processes by which hairpins affect hybridization, including the origin of their unexpectedly large effect on dissociation, we use oxDNA (23–25), a coarse-grained nucleotide-level model of DNA, to simulate and study the systems of Gao *et al*. (1). We reproduce to a good approximation their observed changes in hybridization rates for different hairpin systems. An important advantage of simulation is that one can go beyond reproducing the thermodynamics to probe the reactive trajectories in a high level of detail. In particular, we clarify the detailed microscopic mechanisms by which hairpins change the rates of hybridization and melting of duplexes.

## MATERIALS AND METHODS

### oxDNA model

**Figure 1.**
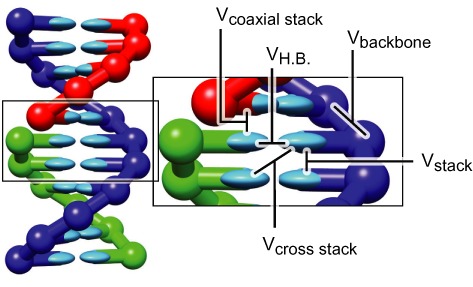
A model DNA duplex, with stabilizing interactions depicted schematically. The backbone sites are shown as spheres, the bases as ellipsoids. Backbone coloring indicates strand identity. All nucleotides also interact with repulsive excluded volume interactions. The coaxial stacking interaction acts like a stacking interaction between bases that are not immediate neighbors along the backbone of a strand. Taken from (26).

The oxDNA model treats each nucleotide as a three-dimensional rigid body. The potential energy of a configuration is given by a sum of pairwise interactions between nucleotides, representing hydrogen-bonding, cross-stacking, coaxial stacking, nearest-neighbor stacking, excluded volume and backbone chain connectivity. These interactions are shown schematically in Figure 1, and discussed in detail elsewhere (23–25). Importantly, attractive interactions depend explicitly on the relative orientations of nucleotides, allowing the anisotropic nature of bases to play a role.

Hydrogen-bonding and stacking interactions drive the formation of duplexes with helical structure from single strands that are relatively disordered. Additionally, oxDNA was fitted at a salt concentration of [Na^+^] = 0.5 M where electrostatics is strongly screened. The model reproduces the thermodynamic, mechanical and structural changes associated with this transition, under high salt conditions. In particular, oxDNA provides a good representation of duplex melting temperatures, melting transitions widths, self-complementary hairpin stability, duplex elastic moduli and the short persistence length of single strands (details are provided in 23–25).

It should be noted that oxDNA neglects several features of DNA structure and interactions due to the high level of coarse-graining. Specifically, the double helix in the model is symmetrical where the grooves between the backbone sites do not have different sizes (i.e. major and minor grooving), and all four nucleotides have the same structure. Additionally, the current version of the model does not include differences between AA and TT dinucleotide steps that have been shown to influence hairpin stability (26). These differences with real DNA mean that oxDNA will not be able to treat phenomena that depend sensitively, for example, on anisotropic elasticity, explicit salt ion effects, or the existence of major and minor grooving. However, these specific properties of DNA are unlikely to be critical to the general arguments we are making about hybridization in the presence of hairpins in this article. Rather, it is the correct treatment of the basic mechanical properties of both single and double strands, together with the basic physics of hydrogen bonding and stacking that determines the emergent physical phenomena we are trying to describe. Overall, the oxDNA model has been highly successful at capturing some of the fundamental biophysics of DNA, including the kinetics of hybridization (27) and toehold-mediated strand displacement (28), as well as providing insights into nanotechnological systems (29).

### Simulation techniques

Thermodynamic results presented in this work are obtained using the virtual-move Monte Carlo (VMMC) algorithm (30,31). Specifically, we use the variant in the appendix of (31). VMMC is an efficient algorithm for sampling from the canonical ensemble for systems consisting of clusters of strongly-interacting particles. To accelerate equilibration, we use biased umbrella sampling (32) to overcome barriers between local minima of free energy. Additionally, we use single histogram reweighting (33) to extrapolate computed free-energies to nearby temperatures, which allows us to compute hairpin and duplex melting curves.

Kinetic results are obtained with molecular dynamics using an Andersen-like thermostat (34), which generates diffusive motion of particles beyond a certain (extremely short) timescale. We use forward flux sampling (FFS) to determine the rates of hybridization (35,36). Before all data collection, we perform lengthy equilibration runs to ensure that the single strands are initialized in thermodynamically representative states. All dynamical simulations were performed at *T*_hyb_ = 20°C. Additional simulation details can be found in the Supplementary Data.

### Experimental systems studied

Using the same strands and terminology as Gao *et al*. (1), we consider probe strands P designed to be complementary to target strands T, which are listed in Table 1. Using the mFold software (37) Gao *et al*. designed the P/T strands to exhibit varying degrees of secondary structure, where the P_0_/T_0_ is designed to have negligible secondary structure in the single-stranded state, and P_3_/T_3_ and P_4_/T_4_ are designed to have stable 3- and 4-base pair hairpins prior to duplex formation. Other than the presence of hairpins, the strands are very similar, having the same length and possessing the same GC-content and similar hybridization free energies relative to unstructured single strands (as predicted by NuPack (38) and measured with oxDNA). It is therefore reasonable to assume that differences in hybridization rates are primarily due to the presence of hairpins.

**Table 1.** Sequences from (1) used in this work. Underlined sequences show hairpin stems that are intended to form by design. Strands with the same subscript are complementary, so if mixed together will form a duplex: P_*n*_ + T_*n*_ → P_*n*_ T_*n*_.

P_0_	3′ −GAG ACT TGC CAT CGT AGA ACT GTT G−5′
P_3_	3′ −TGA CGA TCA TGT CTG CGT GAC TAG A−5′
P_4_	3′ −ACA CGA TCA TGT CTG CGT GAC TAG A−5′
T_0_	3′ −CAA CAG TTC TAC GAT GGC AAG TCT C−5′
T_3_	3′ −TCT AGT CAC GCA GAC ATG ATC GTC A−5′
T_4_	3′ −TCT AGT CAC GCA GAC ATG ATC GTG T−5′

Gao *et al*. performed their experiments at room temperature (20°C), using a ssDNA concentration of 2 μM, and a high salt concentration of [Na^+^] = 0.5 M (1). This high salt concentration (39) is the same as that used for the duplex melting curves to which oxDNA has been parameterized (23), so duplex behavior should be reproduced well. At this salt concentration, electrostatic interactions between nucleotides are screened. These electrostatic interactions oppose DNA structure formation, so DNA nanotechnology usually uses similar high salt concentrations.

## RESULTS AND DISCUSSION

### Single-strand thermodynamics

**Figure 2.**
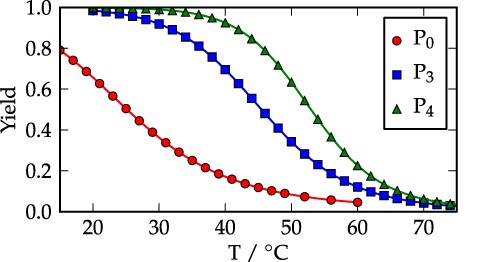
Equilibrium yields of the hairpins versus temperature for the *P* strand in each system. Similar results are found for the *T* strands.

To determine the prevalence of stable secondary structure at room temperature, we calculated the yields of hairpins in the three P strands, shown in Figure 2, using VMMC assisted by umbrella sampling (the results for the T strands are similar). States are designated as hairpins if they possess at least one intra-strand base pair. P_0_ and T_0_ have no well-defined strong secondary structure, although a number of transient hairpin configurations contribute to the ‘hairpin state’. The computed melting temperature of 24°C is only marginally above 20°C, the temperature used for hybridization measurements, and hairpins are only present ∼60% of the time at 20°C. By contrast, the computed melting temperatures for P_3_ and P_4_ secondary structure are approximately 46°C and 54°C, respectively. Both systems are dominated by hairpins with approximate yields of 99.8% for P_4_ and 98.4% for P_3_ at 20°C. These melting curves are also broadly consistent with those measured by Gao *et al*. (1).

**Figure 3.**
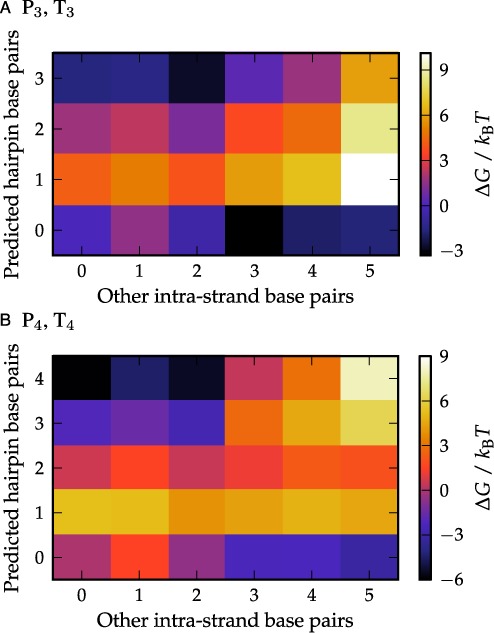
The free-energy profiles of the P_3_ and P_4_ strands are shown in panels (**A**) and (**B**), respectively. The free energy of a particular state relative to the unbound (0,0) state is indicated by the color of the square. The results for complementary T strands are essentially identical.

Free-energy landscapes for these strands computed using oxDNA are plotted in Figure 3. From Figure 3(a) it is clear for the P_3_ and T_3_ strands that the predicted 3-base pair hairpin in the (0, 3) state is low in free energy compared to the state with no secondary structure. However, there exist other significant states of the strands, notably the (3, 0) state, which incorporates several 3-stem hairpins of a similar free energy. We found that this state was dominated by a hairpin with a very long 12-base pair tail. The (4, 0) and (5, 0) states showed pseudoknot behavior, with the strand bending back on itself twice. The (2, 3) state corresponded to the predicted 3-stem hairpin, but with the tails partially hybridized with two base pairs, producing a smaller trailing tail for the structure. These significant hairpin states are shown schematically in Supplementary Figure S2. The presence of multiple stable 3-stem hairpins in oxDNA is consistent with the predictions of NuPack (38). Figure 3(b) shows that the P_4_ and T_4_ strands both show that the predicted 4-base pair hairpin, located at (0, 4), is extremely stable. Also noteworthy is the state located at (2, 4) in Figure 3(b), which consists of the predicted hairpin with two of the base pairs in the loops bonded.

### Duplex thermodynamics

**Figure 4.**
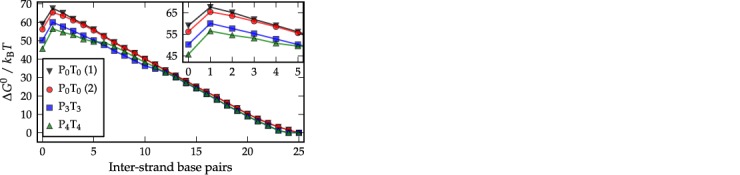
The free-energy profiles for P_0_T_0_, P_3_T_3_ and P_4_T_4_ versus inter-strand base pairs between P and T strands. For P_0_T_0_, curve (1) is obtained when hairpin formation is forbidden, and curve (2) is from a simulation without this restriction. Each system was simulated in a box with a volume of 3.96 × 10^−23^ m^−3^ at *T* = 20°C.

**Figure 5.**
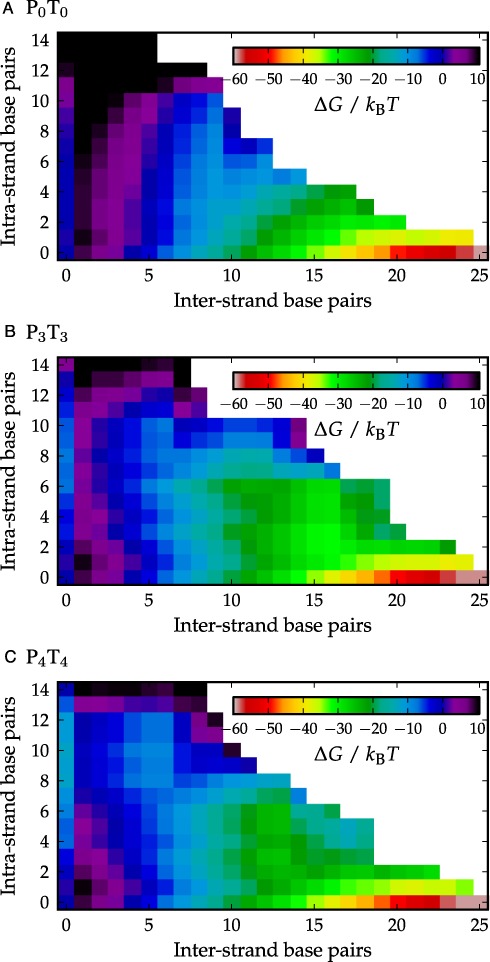
Free energies measured relative to the unbound state containing no intra-strand structure are plotted in panel (**A**) for P_0_T_0_, in panel (**B**) for P_3_T_3_, and in panel (**C**) for P_4_T_4_ as a function of any complementary inter-strand base pair present between the two strands, and any complementary intra-strand base pair present within P or T strands. Black color indicates states which have Δ*G* ≥ 10*k*_B_*T*.

In Figure 4, we plot free-energy landscapes for the three duplex systems as a function of the number of inter-strand base pairs, while in Figure 5 two-dimensional (2D) free-energy landscapes for the P_0_T_0_, P_3_T_3_ and P_4_T_4_ systems are shown as a function of inter- and intra-strand base pairs, respectively. Figure 4(a) clearly shows that hairpin formation in each single strand lowers the free energy of the single-stranded state relative to the fully formed duplex, specifically, by approximately 2.2 *k_B_T*, 8.6 *k_B_T* and 13.4 *k_B_T* for P_0_T_0_, P_3_T_3_ and P_4_T_4_ respectively. If these differences in single-strand stability were to be manifest only in *k*_+_, we would expect hybridization to be slowed by a factor of ∼600 for P_3_T_3_ and by a factor of ∼7 × 10^4^ for P_4_T_4_ relative to P_0_T_0_.

Figure 5 shows 2D free-energy landscapes as a function of both inter- and intra-strand base pairs. For P_0_T_0_, strands that have formed one inter-strand base pair may contain a variety of intra-strand base pairs, largely reflecting the fact that the unbound P_0_ and T_0_ strands were found to contain some transient intra-strand base pairs. There are no obvious partially-bound metastable states in this landscape.

In the P_3_T_3_ case, the hairpins are relatively stable and their effects on the free-energy landscape are more apparent than in the P_0_T_0_ system. For states in which hairpins are not present (the row corresponding to intra-strand base pairs equal to zero), the landscape is essentially identical to that of P_0_T_0_, supporting the suggestion that differences in behavior are mostly due to the hairpins. However, the states with the lowest free energy for a given number of inter-strand base pairs all contain hairpin structure until 13 inter-strand base pairs in the P_3_T_3_ system, unlike in the P_0_T_0_ case. To increase the number of inter-strand base pairs from this point, one or both hairpins must melt. In this landscape, there is some evidence of barriers between intermediate states with and without hairpins, consistent with the existence of metastable states.

Lastly, the P_4_T_4_ free-energy landscape shown in Figure 5(c) clearly shows the presence of stable 4-stem hairpins when up to six inter-strand base pairs are present between the P and T strands. There is also clear evidence of barriers between intermediate states with and without hairpins. As with P_3_T_3_, however, the landscape is almost identical to P_0_T_0_ for the states in which hairpins are absent.

During preliminary simulations, we observed the occasional presence of a pseudoknotted/‘kissing’ complex, illustrated in Supplementary Figure S6, in which the hairpins are still present, but with inter-strand base pairs between both the tail and the loop of the hairpin. This state proved difficult to sample with the order parameter used to generate Figure 5. We therefore ran additional simulations, detailed in Supplementary Section SIII, to measure the stability of the pseudoknotted state relative to the state with inter-strand base pairs between tails only. The results in Supplementary Figure S5 show an additional minimum around (13,8) corresponding to the pseudoknotted state that is less stable than the tail-only state.

### Kinetics

In Table 2 we give the relative hybridization rates for the three systems as simulated by oxDNA compared to those reported by Gao *et al*. (1). Clearly, the slowdown of hybridization rates is comparable in simulation and experiment. We note that for P_4_T_4_ Gao *et al*. observed two regimes with apparently distinct rate constants. Such behavior might be indicative of a long-lived metastable intermediate state. We observe metastable intermediates during our simulations (see Supplementary Section SIV) but they are not sufficiently long-lived to result in deviations from simple second-order kinetics at the strand concentrations used by Gao *et al*., which is unsurprising given the known physics of DNA. However, Gao *et al*. predict simple second-order behavior at lower concentrations with the P_3_T_3_ and P_4_T_4_ duplex formation rates slowed by factors of ∼2 and ∼6 relative to P_0_T_0_. The predictions of oxDNA are quantitatively similar in this second-order regime.

**Table 2.** (Top row) Hybridization (*k*_+_) and melting (*k*_−_) rate constants for P_3_T_3_ and P_4_T_4_ duplexes, all measured relative to P_0_T_0_, for the experiment (1) (*k*_+_ only) and oxDNA. In the case of P_4_T_4_, Gao *et al*. claimed to measure a fast (f) and a slow (s) regime (1) (Bottom row). Listed are the measured rates, *r*_+_, relative to P_0_T_0_ that a state starting from the unbound state goes to a state with one inter-strand base pair (second column), and the measured probabilities, *P*_+_, relative to P_0_T_0_ that a state starting from one inter-strand base pair goes to a full duplex with 25 base pairs (third column). Listed are the ratios of the calculated equilibrium constants measured relative to P_0_T_0_.

Duplex	Experiment $k^{0}_{+}$/*k*_+_	oxDNA $k^{0}_{+}$/*k*_+_	oxDNA *k*_−_/$k_{-}^{0}$
P_0_T_0_	1	1	1
P_3_T_3_	1.8 ± 0.2	2.1 ± 0.3	100 ± 25
P_4_T_4_	6.0 ± 0.3 (f)	10.6 ± 3.4	1934 ± 712
	25.0 ± 1.3 (s)		
	$r^{0}_{\scriptscriptstyle +}$/$r_{\scriptscriptstyle +}^{\vphantom{0}}$ (0 → 1)	$P^{0}_{\scriptscriptstyle +}$/$P_{\scriptscriptstyle +}^{\vphantom{0}}$ (1 → 25)	$K_{\text{eq}}^{0}$/$K_{\text{eq}}^{\vphantom{0}}$
P_0_T_0_	1	1	1
P_3_T_3_	1.85 ± 0.17	1.14 ± 0.19	204 ± 45
P_4_T_4_	2.11 ± 0.11	4.93 ± 0.33	20539 ± 4096

**Figure 6.**
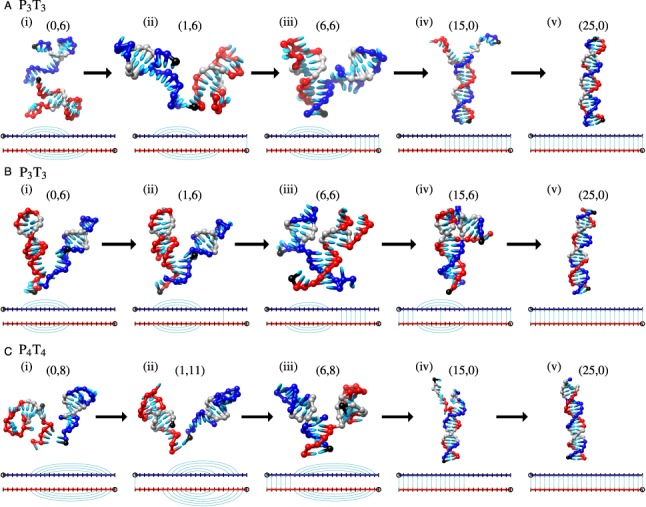
Typical duplex formation pathways for panels (**A**) and (**B**) P_3_T_3_, and panel (**C**) P_4_T_4_. Schematic illustrations below each figure indicate hairpin and duplex base pairs present. In panel (A) one intended and one non-intended 3-stem base pair are present just before and after the association event ((i) and (ii)) but have melted by the time the system has formed 15 inter-strand base pairs (iii). In panel (B) both intended 3-stem base pairs are present just before and after the association event ((i) and (ii)), and are still present even once the system has formed 15 inter-strand base pairs (iv). These hairpins must melt before the duplex can be formed (v). In panel (C) both intended 4-stem base pairs are present in (i)-(iii), but have melted by the time the system has formed 15 inter-strand base pairs (iv). The black nucleotides indicate the 3′ end of the strand. Gray nucleotides indicate the locations of the 3-base pair or 4-base pair hairpins present during the association event. The notation (1,6), for example, refers to 1 inter-strand and 6 intra-strand base pairs in the system.

The overwhelming majority of duplex formation events in oxDNA, both for P_3_T_3_ and P_4_T_4_, follow a pathway in which the strands associate with intact hairpins (about 95% and 99% of configurations for P_3_T_3_ and P_4_T_4_ systems, respectively), as is suggested for P_4_T_4_ in Figure 5(c). A typical pathway for P_3_T_3_, illustrated in Figure 6(a), shows that strands retain their 3-base pair hairpins after initially binding (6(a,i-ii)). These hairpins are typically still present when the two strands contain significant duplex structure (6(a,iii)), but must then melt (6(a,iv)) before proceeding to a full duplex (6(a,v)). Similarly, in the P_4_T_4_ system, up to 6 base pairs can form between the longer tails with no loss of hairpin base pairs (Figure 6(c,iii)). These states, which reside at the local minima at ∼(5, 8) in Figure 5(c), are then blocked by the hairpins from zippering up further. For successful hybridization of P_4_T_4_, one of the hairpins needs to first melt. To proceed further, either the second hairpin must spontaneously melt before the first reforms, or the first strand could open the other hairpin by displacement. Zippering up of the duplex to completion can then follow.

Figure 6(b) exhibits an alternative hybridization pathway for P_3_T_3_ when both strands contain the intended hairpin. In this case, the intermediate structure in Figure 6(b-iv) has formed a four-way junction containing 10 base pairs between the longer tails of the hairpins and 4 base pairs between the shorter tails (and one base pair between the hairpin loops). For this P_3_T_3_ intermediate, the interaction between the longer tails is sufficiently strong that we never see the strands separate rather than forming the full duplex, which can be accomplished either through migration of the four-way junction, where the intra-strand base pairs can be exchanged for inter-strand base pairs at low free-energetic cost, or through melting of the hairpins and zippering up. For P_4_T_4_, however, pseudoknotted intermediate states such as that in Supplementary Figure S6 (which contain inter-strand base pairs between the tails and loops of the hairpins) frequently dissociate, even if 15 inter-strand base pairs are present in total. This difference is because it is topologically unfeasible for the duplex to form without base pairs first being lost. Typically the weaker kissing interactions between the hairpin loops first melt taking the system back to the (5,8) intermediate, and once there the system is more likely to melt than to progress to complete duplex formation. For both P_3_T_3_ and P_4_T_4_, intermediate states are not sufficiently stable within oxDNA to act as extremely long-lived metastable states (see Supplementary Section SIV).

As the hybridization reactions of these strands follow second-order kinetics to a very good approximation in oxDNA at low strand concentrations (see Supplementary Section SIV), and we already know *k*_+_ and Δ*G*^0^, we can use Eq. (1) to infer *k*_−_ directly without additional expensive simulations. The resulting values of *k*_−_ for 3- and 4- base pair hairpins are roughly two and three orders of magnitude larger than for the P_0_T_0_ system, respectively, as shown in Table 2. Consequently, these hairpins primarily influence the transition kinetics by increasing *k*_−_ rather than suppressing *k*_+_, confirming our inference from the experimental data.

Although we have not actually simulated melting trajectories, the principle of microscopic reversibility applies and the ensemble of melting trajectories is identical to the ensemble of formation trajectories in reverse. Thus our prior analysis can be applied to the melting trajectories. Most importantly, we conclude that hairpins are not only present after the strands come into contact during assembly, but also prior to strand separation during melting. As one can see from Figure 4, once the duplex starts to melt, hairpins can form and stabilize the partially melted states relative to the fully formed duplex, leading to significantly lower free-energy barriers for melting for P_3_T_3_ and P_4_T_4_ than for P_0_T_0_ (by 6 and 10 *k_B_T*, respectively). As a consequence, the system has a much higher rate of detachment than it would in the absence of hairpins, explaining the inferred increase in *k*_−_. We note that previous experimental studies have suggested either that hairpins might not fully open prior to hybridization (40) or that they can form during dissociation (41). Our explanation of the fact that hairpins act primarily to increase dissociation is consistent with these results.

Having confirmed that oxDNA reproduces and explains the relatively weak reduction in *k*_+_ (and consequent strong increase in *k*_−_) due to hairpins seen by Gao *et al*., we now analyze the factors contributing to the reduction of *k*_+_ in more detail. Possible contributions to the decrease in *k*_+_ include a reduction in the initial association rate due to fewer bases being accessible for bonding, and also a reduction in the probability that a partially hybridized structure leads to a full duplex. To determine their relative roles, we decomposed the hybridization rate for each system into the rate of initial association, and the probability that an initial association leads to the successful formation of the full duplex. Table 2 shows that the presence of the hairpins slows the rate of association for P_3_T_3_ and P_4_T_4_ by roughly a factor of two in each case when compared to the hairpin-free system. Additionally, the probability that the first base pair between the two strands leads to a duplex is roughly the same for P_0_T_0_ and P_3_T_3_ systems, while for P_4_T_4_, the initial base pair is ∼5 times less likely to lead to a full duplex when compared to the hairpins-free case.

**Figure 7.**
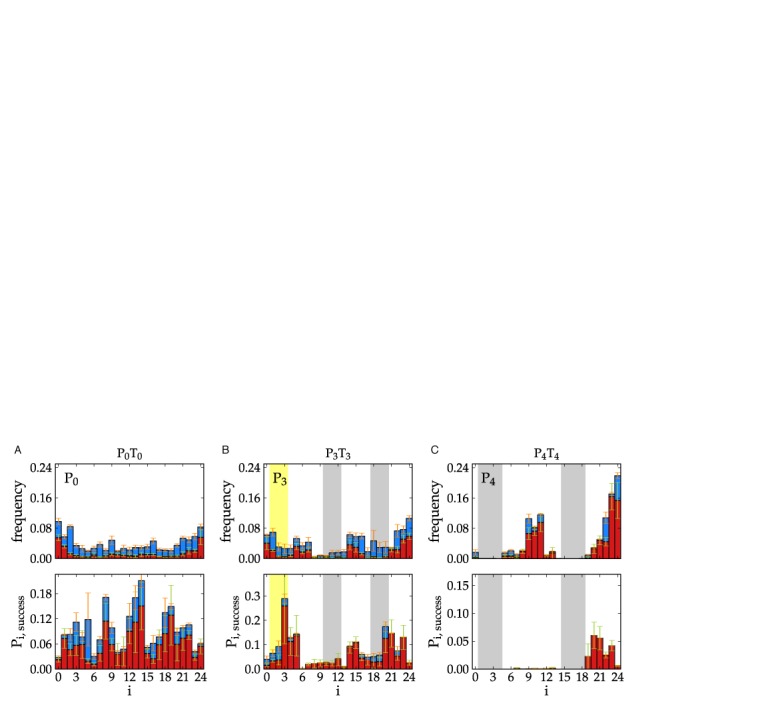
The frequencies of attachment locations for configurations that formed an initial association base pair as a function of base pair index on the P strand are plotted in the top panel in panel (**A**) for P_0_T_0_, in panel (**B**) for P_3_T_3_, and in panel (**C**) for P_4_T_4_. The probability that said base pairs lead to a duplex are plotted in the bottom panels in panel (A) for P_0_T_0_, in panel (B) for P_3_T_3_, and in panel (C) for P_4_T_4_. In each panel, red color indicates contributions from correctly aligned duplex base pairs between the two strands, while blue color indicates contributions from misaligned base pairs between the two strands. The yellow region indicates the location of a non-intended 3-base pair hairpin, which pairs with bases at locations 10-12. The grayed out regions for P_3_ indicate the intended 3-base pair hairpin stem, while the grayed out regions for P_4_ indicate the 4-base pair hairpin stem. For P_4_T_4_, nucleotides 5-14 are within the loop of the hairpin, and nucleotides 19-24 are a dangling tail.

To analyze the initial association events further, in Figure 7 we plot the frequency with which initial inter-strand base pairs form at different points on the P strand, for P_0_T_0_ (Figure 7(a)), P_3_T_3_ (Figure 7(b)) and P_4_T_4_ (Figure 7(c)). For P_0_T_0_, there is little systematic variation, except for a bias towards forming base pairs at the strand ends (27). For P_4_T_4_, however, the bases involved in the hairpin stem never form the initial base pairs. Bases that are adjacent to the stem are also harder to access, meaning that initial base pairs tend to form near the apex of the hairpin loop or in a dangling single-stranded tail that is present in both hairpins of the P_4_T_4_ system. This reduction in the number of feasible initial binding sites is also evident from Figure 4 where the free-energy barrier separating bound and unbound states increases by ∼0.6*k*_B_*T* and ∼1.6*k*_B_*T* for P_3_T_3_ and P_4_T_4_, respectively, as compared to the same quantity for P_0_T_0_.

Unlike in the P_4_T_4_ case where two 4-base pair hairpins dominate, there may be different combinations of 3-base pair hairpins present in the P_3_ and T_3_ strands during association, as was discussed in Section IIIA. Because of this, the plots for P_3_ and T_3_ shown in Figure 7(b) are less revealing of the effects the hairpins have on hybridization rates than the equivalent plots for P_4_T_4_. Overall, Figure 7(b) illustrates that initial base pairs between P_3_ and T_3_ strands can occur at any binding site, and successful binding events can come from any initial base pair, but there seems to be a bias away from the center of the strand because sites 10-12 (gray color in Figure 7(b)) may form base pairs with the bases at sites 1-3 (yellow color in Figure 7(b)) or 18-20 (gray color in Figure 7(b)).

The bottom panel in Figure 7(a) shows that for P_0_T_0_ all initial binding sites have a non-zero chance of leading to the full duplex (G-C base pairs being more likely to succeed). By contrast, the bottom panel in Figure 7(c) shows that only the bases in the single-stranded tails of the P_4_ and T_4_ hairpins have a reasonable probability of subsequently leading to full duplex formation (and even for these bases, the success rate is lower than that typical of the P_0_T_0_ case). Thus, P_4_T_4_ strands are unlikely to form a full duplex if the initial binding occurs between the hairpin loops. Although up to 10 base pairs could potentially form between the loops, such ‘kissing complexes’ are topologically and geometrically frustrated and are free-energetically much less stable than the equivalent duplex with the same number of base pairs (42). They are therefore much more likely to fail than to succeed. Even if one of the hairpins were to open, displacement of the other stem from the internal toehold (i.e. the loop) is significantly harder than for an external toehold (18).

**Figure 8.**
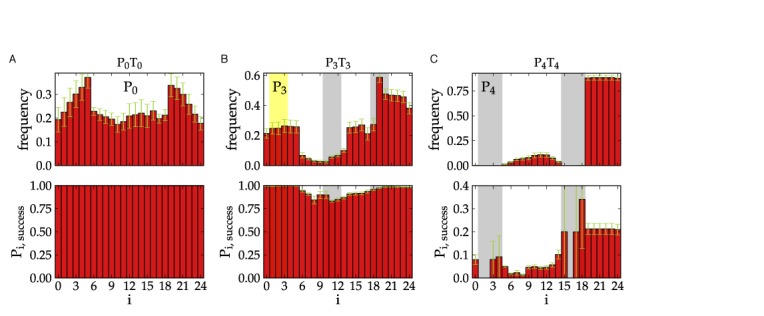
The frequencies of attachment locations for configurations that have formed six inter-strand base pairs as a function of base pair index on the P strand are plotted in the top panel in panel (**A**) for P_0_T_0_, in panel (**B**) for P_3_T_3_, and in panel (**C**) for P_4_T_4_. The probability that said base pairs lead to a duplex are plotted in the bottom panels in panel (A) for P_0_T_0_, in panel (B) for P_3_T_3_, and in panel (C) for P_4_T_4_. Similar to Figure 7, the yellow region indicates a the location of a non-intended 3-base pair hairpin, which pairs with bases at locations 10-12. The grayed out regions for P_3_ indicate the intended 3-base pair hairpin stem, while the grayed out regions for P_4_ indicate the 4-base pair hairpin stem. Sites 5-14 are within the loop of the hairpin, and sites 19-24 are a dangling tail.

The 3-base pair hairpins offer less impediment to successful duplex completion for two reasons. Firstly, a 3-base pair hairpin melts considerably faster than a 4-base pair hairpin. Secondly, the P_3_T_3_ system is able to form more base pairs (e.g. ten base pairs between the longer tails when both strands have the intended hairpins) before being blocked by the hairpins (Figure 6(iv)), and once the system has reached this stage dissociation is very unlikely (Figure 8(b)). By contrast for P_4_T_4_, even once the partially hybridized system contains at least 6 inter-strand base pairs, there is still an 80% probability that the system will dissociate, as is illustrated in Figure 8(c), and is due to increased stability of the hairpins and the reduced stability of the metastable states prior to hairpin disruption in P_4_T_4_. The earlier loss of hairpin base pairs for P_4_T_4_ is evident from Figure 4, where the profile for P_4_T_4_ approaches that for P_0_T_0_ after fewer inter-strand base pairs.

### Effects due to mis-aligned inter-strand base pairs

In Figure 7 the initial association base pairs, and their probabilities of success, have been separated into contributions from correctly aligned base pairs, and mis-aligned base pairs (Supplementary Tables SIV and SV tabulate the success probabilities between interfaces as hybridization proceeds). The probabilities of the strands forming two inter-strand base pairs in the simulations clearly shows that the P_0_T_0_ system is less successful than the P_3_T_3_ and P_4_T_4_ systems at forming subsequent base pairs after the first has formed, where P_0_T_0_, P_3_T_3_ and P_4_T_4_ systems have a 14%, 20% and 24% chance of successfully forming two inter-strand base pairs. The effect is due to the higher incidence of mis-aligned base pairs forming compared to correctly aligned base pairs (see Figure 7, top panel).

Mis-aligned base pairs are less likely to lead to more inter-strand base pairs because neighboring bases are not likely to be complementary. Furthermore, the P_3_T_3_ system is more likely to form mis-aligned base pairs than the P_4_T_4_ system. Generically, two strands containing *N* binding sites, which contain negligible secondary structure, can be bound in ∼*N*^2^ number of possible ways. For P_3_T_3_ and P_4_T_4_ systems there are less ways for the strands to mis-align during association due to larger hairpins present in the two systems obscuring binding sites. This is evident from the bottom panels in Figure 7, which shows a decreasing likelihood that mis-aligned base pairs lead to a full duplex. For the P_4_T_4_ system, mis-aligned base pairs almost never lead to a full duplex, but rather lead to strand separation. The increased probability of P_3_T_3_ configurations forming two inter-strand base pairs after the first association base pair has formed compared to P_0_T_0_ is offset by a lower probability of subsequently forming six base pairs (because of the blocking effects of the hairpins). The combination of these two effects gives rise to very similar probabilities of achieving a full duplex given 1 base pair for P_0_T_0_ and P_3_T_3_, as noted in Table 2.

## CONCLUSION

In summary, we have shown numerical evidence that although hairpins slow hybridization reactions, the rate reduction can be far smaller than might naively be expected from the change in stability of the duplex, Δ*G*^0^. Our results are in quantitative agreement with Gao *et al*. (1), supporting our inferences based on their experimental data, which we have shown to be consistent with the basic physics of DNA as represented by oxDNA. The result of this observation is that hairpins can increase unbinding rates far more than they decrease binding rates.

In addition to reproducing and confirming the above effect, we have provided a microscopic mechanism. Hybridization reactions do not proceed through the opening of hairpins and subsequent binding of strands, but rather initial contacts between strands form with the hairpins in place. This implies that hairpins form during the melting process, stabilizing the partially-melted states and enhancing detachment rates.

Hairpins slow down the hybridization process both because potential binding sites are hidden by the secondary structure, and because the hairpins reduce the probability of initial base pairs leading to a full duplex by interfering with the ‘zippering’ up of the strands. The stability of the hairpin stem, which is influenced by both sequence and length, is likely to be a major factor determining the magnitude of the latter effect, e.g. longer stems are generally harder to open up. In addition, the position of the hairpin can also play an important role. In particular, if the single stranded tails are long, they are much less likely to dissociate prior to hairpin opening and hence the influence of the hairpins on *k*_+_ is reduced. Long hairpin loops could also potentially play a similar role, but only if particularly stable kissing complexes can form (i.e. with a lifetime comparable to or greater than the hairpin stems: in none of the systems studied do kissing complexes play a dominant role in association). Hairpins containing long stems, short tails and small loops would therefore be expected to maximize the reduction in *k*_+_.

**Figure 9.**
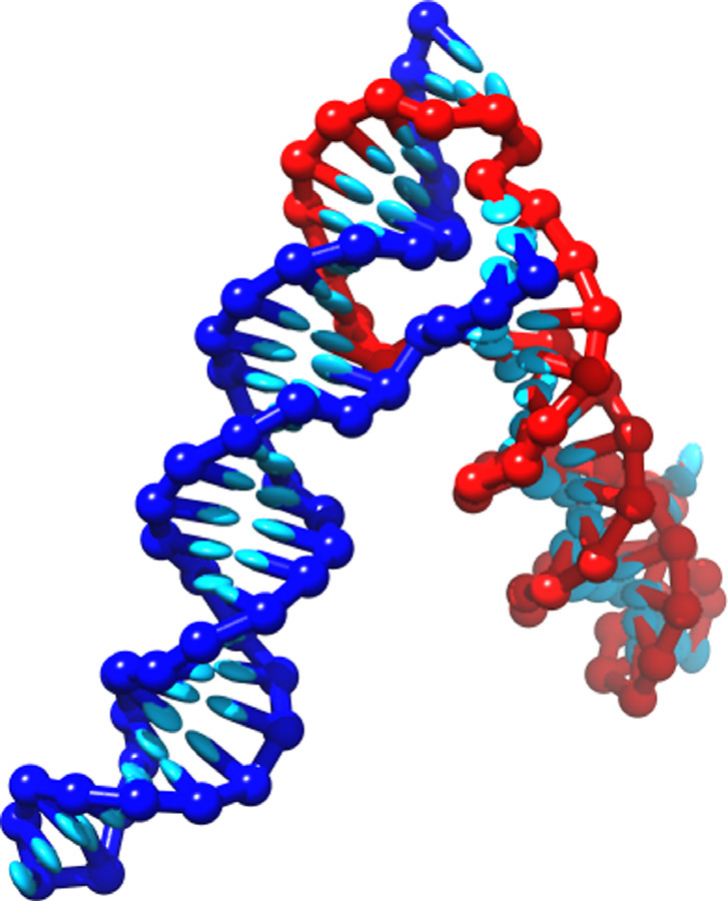
Two long hairpins form a four-way junction after fraying base pairs at the end of their stems. In total, the structure contains 14 inter-strand base pairs and 27 intra-strand base pairs. Duplex formation can proceed through a process in which intra-molecular base pairs are exchanged for inter-molecular base pairs.

Although *k*_−_ is greater for P_4_T_4_ than P_3_T_3_ and P_0_T_0_, melting is still very slow at room temperature. Nevertheless, using longer hairpin stems or shorter duplexes should lead to experimentally measurable changes in *k*_−_ with hairpin content. Furthermore, our results show that hairpins may be less effective as a deliberate design to prevent hybridization than might have been hoped for (and that small unintended hairpins present only a minor impediment to assembly processes in DNA nanotechnology because so much of the change in Δ*G*^0^ is absorbed into *k*_−_, even if it remains small). As mentioned above, carefully designed hairpins with no tails, short loops and long stems should maximize the reduction in *k*_+_. However, even in these cases, hairpins are likely to have a much larger effect on *k*_−_ than *k*_+_ (although the effect on *k*_+_ may still be significant). Such hairpins are unlikely to form duplexes by first melting their entire stem. Instead, if a few base pairs fray in each hairpin, the strands can form a four-way junction as illustrated in Figure 9. From this point, intra-molecular base pairs can be exchanged for inter-molecular base pairs at very low free-energetic cost, and duplex formation can happen through a process analogous to branch migration in Holliday junctions. Similar migrating four-way junctions have been postulated in experimental studies of hairpin/duplex systems (40,41). Considering the process in reverse, large partially-formed hairpins at four-way junctions will help to compensate for disrupted inter-strand base pairs, stabilizing partially melted states and hence enormously increasing *k*_−_. A partial solution to this problem would be to add extra base pairs, which will be mismatches in the final duplex, to the stems of hairpins. These mismatches will suppress the displacement and four-way branch migration pathways identified here.

## SUPPLEMENTARY DATA

Supplementary Data are available at NAR Online.

SUPPLEMENTARY DATA
