# Supplementary material for: DNA hairpins destabilize duplexes primarily by promoting melting rather than by inhibiting hybridization
Source: Nucleic Acids Res. 2015 Jun 8;43(13):6181–90. doi: 10.1093/nar/gkv582 (PMC4513862; doi:10.1093/nar/gkv582)
Supplement: SUPPLEMENTARY DATA [file supp_43_13_6181__index.html]

DNA hairpins destabilize duplexes primarily by promoting melting rather than by inhibiting hybridization — DNA hairpins destabilize duplexes primarily by promoting melting rather than by inhibiting hybridization — SUPPLEMENTARY DATA 

# DNA hairpins destabilize duplexes primarily by promoting melting rather than by inhibiting hybridization

## SUPPLEMENTARY DATA

- SUPPLEMENTARY DATA
