# Supplementary material for: DNA hairpins destabilize duplexes primarily by promoting melting rather than by inhibiting hybridization
Source: Nucleic Acids Res. 2015 Jun 8;43(13):6181–90. doi: 10.1093/nar/gkv582 (PMC4513862; doi:10.1093/nar/gkv582)
Supplement: SUPPLEMENTARY DATA [file supp_gkv582_nar-00565-f-2015-File004.pdf]

# Supplemental material for "DNA hairpins primarily promote duplex melting rather than inhibiting hybridization"

John S. Schreck,[1,*] Thomas E. Ouldridge,[2,3,†] Flavio Romano,[1] Petr Šulc,[4] Liam Shaw,[5] Ard A. Louis,[5] and Jonathan P. K. Doye[1,‡]

[1] *Physical and Theoretical Chemistry Laboratory, Department of Chemistry, University of Oxford, South Parks Road, Oxford OX1 3QZ, United Kingdom*
[2] *Rudolf Peierls Centre for Theoretical Physics, University of Oxford, 1 Keble Road, Oxford OX1 3NP, United Kingdom*
[3] *Department of Mathematics, Imperial College, 180 Queen's Gate, London, SW7 2AZ, United Kingdom*
[4] *Center for Studies in Physics and Biology, The Rockefeller University, 1230 York Avenue, New York, NY 10065, USA*
[5] *Rudolph Peierls Centre for Theoretical Physics, University of Oxford, 1 Keble Road, Oxford OX1 3NP, United Kingdom*

## CONTENTS

SI. oxDNA Model Details 1

SII. Simulation Methods 2
- A. Thermodynamics 2
  - 1. Virtual Move Monte Carlo 2
  - 2. Umbrella Sampling 2
  - 3. Single Histogram Reweighting 3
- B. Kinetics 3
  - 1. Molecular Dynamics 3
  - 2. Forward Flux Sampling 3

SIII. Simulation Protocols 4
- A. Thermodynamics 4
  - 1. Single-strand Thermodynamics 4
  - 2. Duplex Thermodynamics 5
- B. Kinetics 6
  - 1. FFS Simulation Details 6
  - 2. Order Parameter Used in FFS Simulations 6
  - 3. Initial Equilibration of Single-strand States 6
  - 4. Second-order Kinetics Approximation 6

SIV. Results 7
- A. Thermodynamics 7
  - 1. Single-strand Thermodynamics 7
  - 2. Duplex Thermodynamics 8
- B. Kinetics 8
  - 1. Considerations of the Kinetic Intermediate States 9
  - 2. Additional Results for Hybridization Pathways 10

References 10

* john.schreck@chem.ox.ac.uk
† t.ouldridge@imperial.ac.uk
‡ jonathan.doye@chem.ox.ac.uk

## SI. OXDNA MODEL DETAILS

OxDNA and its interaction potentials have been described in detail elsewhere [1–3]. The model represents DNA as a string of nucleotides, where each nucleotide (sugar, phosphate and base group) is a rigid body with three interaction sites. The potential energy of the system can be decomposed as

$$V = \sum_{\langle ij \rangle} \left( V_{\text{b.b.}} + V_{\text{stack}} + V^{'}_{\text{exc}} \right) + \sum_{i,j \notin \langle ij \rangle} \left( V_{\text{HB}} + V_{\text{cr.st.}} + V_{\text{exc}} + V_{\text{cx.st.}} \right), \quad \text{(S1)}$$

where the first sum is taken over all nucleotides that are nearest neighbors on the same strand and the second sum comprises all remaining pairs. The interactions between nucleotides are schematically shown in Fig. 1 in the main article. The backbone potential $V_{\text{b.b.}}$ is an isotropic spring that imposes a finite maximum distance between backbone sites of neighbors, mimicking the covalent bonds along the strand. The hydrogen bonding ($V_{\text{HB}}$), cross stacking ($V_{\text{cr.st.}}$), coaxial stacking ($V_{\text{cx.st.}}$) and stacking interactions ($V_{\text{stack}}$) are anisotropic and explicitly depend on the relative orientations of the nucleotides as well as the distance between the relevant interaction sites. This orientational dependence captures the planarity of bases, and helps drive the formation of helical duplexes. The coaxial stacking term is designed to capture stacking interactions between bases that are not immediate neighbors along the backbone of a strand. Base and backbone sites also have excluded volume interactions $V_{\text{exc}}$ and $V^{'}_{\text{exc}}$.

Hydrogen-bonding interactions are only possible between complementary (A-T and C-G) base pairs. In the sequence-dependent parameterization that we use for all simulations, the strengths of interactions $V_{\text{stack}}$ and $V_{\text{HB}}$ further depend on the identity of the bases involved [3]. Interactions were fitted to reproduce melting temperatures and transition widths of oligonucleotides, as predicted by SantaLucia's nearest-neighbor model [4]. Note that our approach is significantly more complex than the nearest-neighbor model. We simply treat the latter as a

high-quality fit to experimental data. For the purpose of parametrization, structural and mechanical properties of both double- and single-stranded DNA are also taken into account in the fitting procedure. In DNA the double helical structure emerges because there is a length-scale mismatch between the preferred inter-base distance along the backbone, and the optimal separation of bases when stacking. It is exactly this feature that drives the helicity of oxDNA, rather than an imposed natural twist on the backbone. Overall, the emphasis in our derivation of oxDNA was on physics relevant to the duplex formation transition. As discussed in the main text, oxDNA has been extensively tested for other DNA properties and systems to which it was not fitted. Our success in describing all these phenomena gives us confidence to use it to study the dynamics of hybridization in the presence of hairpins.

## SII. SIMULATION METHODS

### A. Thermodynamics

#### 1. Virtual Move Monte Carlo

A standard approach for calculating thermodynamic properties of computational models is the Metropolis algorithm [5]. A drawback with this approach is that only moving single particles at a time results in slow equilibration for systems with strong attractions. This is true for DNA strands, where collective diffusion is strongly suppressed if nucleotides are moved individually. Simulations can be made more efficient by using the Virtual Move Monte Carlo (VMMC) algorithm proposed by Whitelam and Geissler which allows for collective diffusion using cluster moves of particles [6]. Specifically, we use the variant presented in the appendix of Ref. 6. Initially a particle is selected, and a move is chosen at random as in the Metropolis algorithm. The particle's neighbors are then added to a co-moving'cluster' with probabilities determined by the energy changes that would result from the move. Consequently, multiple particles tend to move at once. To use VMMC, we must select 'seed' moves of a single particle. For all VMMC simulations reported here, the seed moves were:

- Rotation of a nucleotide about its backbone site, with the axis chosen uniformly on the unit sphere and the angle drawn from a normal distribution with a mean of zero and a standard deviation of 0.22 radians.

- Translation of a nucleotide, where the displacement along each Cartesian axis is drawn from a normal distribution with a mean zero and a standard deviation of 0.15 simulation units of length (0.1277 nm).

To improve efficiency, if the algorithm generates a cluster move involving more than 7 particles the move is automatically rejected.

#### 2. Umbrella Sampling

An important concept is that of a reaction coordinate (or order parameter) $Q$, which groups together microstates of a system that share some macroscopic property (for example, all configurations of strands with a certain number of base pairs). The free-energy profile as a function of $Q$ can provide useful information about the reaction, provided an appropriate choice has been made. Free-energy barriers can make certain regions of configuration space hard to reach, which prevents efficient sampling of all of the states of interest. The free-energy landscape can be artificially flattened by weighting states with different values of $Q$ appropriately, a technique known as umbrella sampling [7]. Thermodynamic properties of the system can then be extracted from simulations by unweighting the resulting distributions.

In particular, for an unweighted simulation a particular microstate with coordinates $\mathbf{q}^N$ and energy $E(\mathbf{q}^N)$ is sampled with probability

$$P(\mathbf{q}^N) \propto e^{-\beta E(\mathbf{q}^N)}. \tag{S2}$$

The equilibrium average of some variable $A(\mathbf{q}^N)$ is then given by the sum over all states, weighted by their Boltzmann factors:

$$\langle A \rangle = \frac{\int A(\mathbf{q}^N) e^{-\beta E(\mathbf{q}^N)} d\mathbf{q}^N}{\int e^{-\beta E(\mathbf{q}^N)} d\mathbf{q}^N}. \tag{S3}$$

By applying a weighting $w = w(Q(\mathbf{q}^N))$ to each value of the order parameter, we change the sampling frequency to

$$P_w(\mathbf{q}^N) \propto w(Q(\mathbf{q}^N)) e^{-\beta E(\mathbf{q}^N)}. \tag{S4}$$

where the subscript $w$ indicates a property of the weighted system. So we can artificially ensure that our simulation samples all states equally by making $P_w$ constant for all microstates. Equilibrium thermodynamic properties are then obtained by unbiasing afterwards, as can be seen by rewriting Eq. (S3) as follows:

$$\langle A \rangle = \frac{\int \frac{A(\mathbf{q}^N)}{w(Q(\mathbf{q}^N))} w(Q(\mathbf{q}^N)) e^{-\beta E(\mathbf{q}^N)} d\mathbf{q}^N}{\int \frac{1}{w(Q(\mathbf{q}^N))} w(Q(\mathbf{q}^N)) e^{-\beta E(\mathbf{q}^N)} d\mathbf{q}^N} \tag{S5}$$

$$= \frac{\langle A/w \rangle_w}{\langle 1/w \rangle_w}. \tag{S6}$$

Throughout this article, it makes sense to use the number of base pairs in our definition of $Q$. This is the usual choice for studying hybridization processes.

### 3. Single Histogram Reweighting

To determine melting temperatures for structures, we implemented the temperature extrapolation method known as single histogram reweighting, based on the method introduced by Ferrenberg and Swensden [8]. The method of single histogram reweighting allows extrapolation of results from simulations at a particular temperature $T_0$ to other temperatures [8]. States of the system are grouped by their value of some quantity $A$ and their energy $E$, so a histogram $p(A, T_0, E)$ can be produced. The temperature-independent density of states $\Omega(A, E)$ can then be inferred via

$$p(A, T_0, E) \propto \Omega(A, E)e^{-\beta_0 E} \tag{S7}$$

where $\beta_0 = 1/RT_0$. The proportionality constant is unknown because we can only ever know the relative ratios of states in our simulations. $\Omega(A, E)$ can then be used to calculate the average value of $A$ at any temperature $T$ by integrating over all possible states:

$$\langle A(T)\rangle = \frac{\int\int A\Omega(A, E)e^{-\beta E}dEdA}{\int\int \Omega(A, E)e^{-\beta E}dEdA}. \tag{S8}$$

We can rewrite this using Eq. (S7) as

$$\langle A(T)\rangle = \frac{\int\int Ap(A, T_0, E)e^{-(\beta_0-\beta)E}dEdA}{\int\int p(A, T_0, E)e^{-(\beta_0-\beta)E}dEdA}. \tag{S9}$$

We point out that our potential energy function (Eq. (S1)) depends explicitly on the temperature through $V_{stack}$. It is straightforward to extend Eqs. (S8) and (S9) to our case. It must be pointed out that the extrapolation can only go so far from the temperature $T_0$ because sampled states will not be representative of the dominant states at other temperatures. For this reason we explicitly calculate free energies at $T = 20°$C for the main data of the manuscript and in subsequent sections here. We only use histogram reweighting for melting curves in the vicinity of the $T_m$ for a particular system, which are of less importance.

In all VMMC simulations employing umbrella sampling (for single strand and double strand systems), we estimated the error of the computed relative free energies corresponding to different states by computing the standard error of the mean value of multiple independent simulations. The details of each simulation, including the order parameters used as well as the number of independent simulations, are discussed in Section SIII A.

## B. Kinetics

### 1. Molecular Dynamics

Kinetic simulations were performed using an Anderson-like thermostat, similar to the one described in appendix A of Ref. 9. The Newtonian equations of motion for the system are integrated by Verlet integration [10] with a discrete time-step $\delta t$, so that the positions, velocities, orientations, and angular velocities of the nucleotides are recalculated at each time-step. This alone would give the DNA strands constant energy and cause ballistic motion. In reality, DNA in a solvent is being bombarded by water particles and thus undergoes Brownian motion. To model Brownian motion, the velocity of each nucleotide is resampled with a probability $p_v = 0.02$ from a Maxwell-Boltzmann distribution at the temperature of the solvent every 103 time steps. The algorithm also resamples angular velocities with a different probability $p_\omega = 0.0067$. The solvent thus acts as a large heat bath at a fixed temperature, ensuring that the simulated system samples from the canonical ensemble. On time scales longer than $N_{Newt}\delta t/p_v$, where $\delta t$ is the integration time step, the dynamics is diffusive. We choose $\delta t = 1.52 \times 10^{-14}$s for all dynamics simulations in this study. In oxDNA this time step gives a diffusion constant $D_{sim}$ for a 14-mer duplex that is about 100 times higher than experimental measurements [11] of $D_{exp} = 1.19 \times 10^{-10}\text{m}^2\,\text{s}^{-1}$.

This artifical increase in $D_{sim}$ is a common procedure for coarse-grained models where higher diffusion constants can be used to accelerate diffusion. Accelerated diffusion can also speed up certain processes by smoothing out, on a microscopic scale, energy profiles [12]. This can be advantageous because it means simulations utilizing coarse-grained models can be used to study more complicated systems. In a previous study using oxDNA, the hybridization kinetics of a non-repetitive sequence was considered [13]. In that study it was shown that using higher friction constants (smaller diffusion coefficients) in simulations utilizing Langevin dynamics at 300K slowed down hybridization, but did not otherwise qualitatively affect the results. In particular, the tendency for initial base pairs to melt away rather than lead to a full duplex was found to be preserved. Our systems are similar to those studied in Ref. 13, possessing similar numbers of total base pairing between the strands, and using a similar simulation temperature. Additionally, many approximations of real DNA have already been made in the construction of the oxDNA model, and we expect that running simulations with a diffusion coefficient that is larger than the experimentally measured value should preserve the effects that hairpins in single strands have on the relative hybridization and dissociation rates.

### 2. Forward Flux Sampling

'Brute force' dynamics simulations using an Anderson-like thermostat are not efficient enough to generate a representative ensemble of trajectories that start in the single-stranded state and end in the duplex state. Thus, we resorted to using Forward Flux Sampling (FFS) to more efficiently calculate fluxes between local free-energy

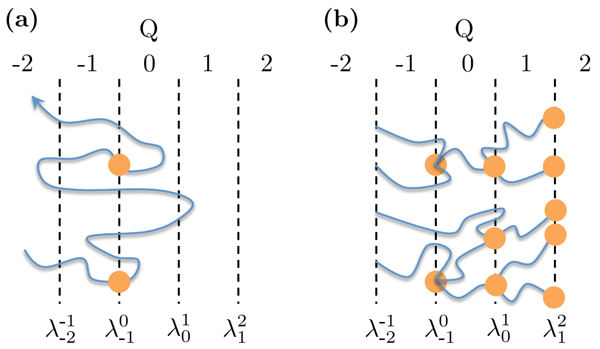


FIG. S1. (Color online) (a) Schematic illustration of the interfaces involved in flux generation. The flux is initially measured across the interface $\lambda^0_{-1}$. The orange dots indicate that a crossing by a trajectory contributes to the flux. These are also the states that are used to launch successive stages of the simulation. (b) In direct FFS, large numbers of configurations are randomly selected from the set that successfully crossed the interface $\lambda^0_{-1}$, and the probability of subsequently crossing the $\lambda^1_0$ interface (rather than returning to $Q = -2$) is measured. The process is then iterated over successively chosen interfaces until reaching $Q_{\max}$. Figure adapted from Ref. 13.

minima as well as sample the transition pathways. The term 'flux' from (meta)stable state A to state B has the following definition:

*Given an infinitely long simulation in which many transitions are observed, the flux of trajectories from A to B is $\Phi_{AB} = N_{AB}/(\tau f_A)$, where $N_{AB}$ is the number of times the simulation leaves A and then reaches B, $\tau$ is the total time simulated, and $f_A$ is the fraction of the total time simulated for which state A has been more recently visited than state B.*

FFS requires use of an order parameter, $Q$, which provides a descriptive measure of the extent of the reaction between states A and B. Additionally, the order parameter must be chosen such that non-intersecting interfaces $\lambda^Q_{Q-1}$ can be drawn between consecutive values of $Q$. At the beginning of an FFS implementation, a brute force simulation is run starting from states described by $Q$ = -2, and the flux of trajectories crossing the surface $\lambda^0_{-1}$ is measured. The total flux of trajectory from $Q$ = -2 to another free-energy minimum $Q = Q_{max}$ can be calculated as the flux of trajectories crossing $\lambda^0_{-1}$ multiplied by the probability that trajectories subsequently reach $Q = Q_{\max}$, all before returning to $Q$ = -2. The probability can be factorized as

$$P\left(\lambda^{Q_{\max}}_{Q_{\max}-1}|\lambda^0_{-1}\right) = \prod_{Q=1}^{Q_{\max}} P\left(\lambda^Q_{Q-1}|\lambda^{Q-1}_{Q-2}\right). \quad \text{(S10)}$$

The first term in the product on the right-hand size of Eq. S10 is calculated by loading random configurations that have just crossed $\lambda^0_{-1}$, which are used to estimate $P(\lambda^1_0|\lambda^0_{-1})$. The process is then iterated for successive interfaces, and the flux as well as the trajectories that successfully reach $Q_{max}$ from the distribution of pathways can be measured.

We estimated the random error in the FFS simulations in the following way. In Table 1 in the main text we report the mean value for the hybridization rates from 5 identical and independent implementations of FFS for each system. The error reported for each system is the standard error of the mean value. In Tables SIII, SIV, and SV, we report the mean and the standard error of the mean for each individual interface. Each calculation of the flux in the three systems was repeated 240 times in total (48 simulations were used to obtain the flux in each independent calculation of the rate), while the probability of crossing interface $\lambda^Q_{Q-1}$ was computed from the 5 independent calculations of the rate.

## SIII. SIMULATION PROTOCOLS

In this section we discuss the implementation of the algorithms of Section SII for both single-stranded and duplex systems. As mentioned in Section SII B 1, we simulated the three duplex systems using molecular dynamics and VMMC simulations. Unless otherwise stated, the temperature in a simulation was taken to be $T = 20°$C, which is the same temperature used by Gao *et al.* in the experiments. Additionally, for simulations of the duplex systems, we used a simulation box with a volume of $3.96 \times 10^{-23}$m$^3$ which corresponds to a concentration of 42 µM, and is 21 times larger than the experimental concentrations of 2 µM used for each system. We also used two types of order parameter in the simulations that we combined into multi-dimensional order parameters, which are discussed individually for each simulation in the sections to follow. Specifically, a 'distance order parameter' measures the minimum distance between hydrogen-bonding sites over correct pairs of bases in the two strands. A 'bonds' order parameter measures the total number of base pairs, which can be specified to be intra- or inter-strand base pairs. The definition of a bonded base pair in our simulations is two bases with a hydrogen bonding energy below 0.596 kcal mol$^{-1}$. This value for the selected cutoff corresponds to about 15% of typical hydrogen-bond energy.

### A. Thermodynamics

#### 1. *Single-strand Thermodynamics*

Melting properties for "monomers" (secondary structure of isolated strands) can be calculated from $\Phi$, the ratio of bound to unbound states in a simulation of a single strand. For self-interacting strands, the fraction of folded states in a hypothetical bulk system is concentration-

independent and can be inferred from

$$f_{\text{bulk}}^{\text{mon}} = f_{\text{sim}} = \frac{\Phi}{1+\Phi}. \tag{S11}$$

The melting temperature is taken to be the point where $f_{\text{bulk}}^{\text{mon}} = 1/2$.

We determined the approximate location of the melting temperatures of the hairpins to ensure that they were stable at $T = 20°\text{C}$. OxDNA has been shown to reproduce the dependence of hairpin melting temperature on stem-length and loop-length [1]. We ran 10 independent VMMC simulations for $6.3 \times 10^{10}$ steps at temperatures of $T = 20°\text{C}$ for both the $P_0$ and $T_0$ strands, $1.1 \times 10^{11}$ and $8.8 \times 10^{10}$ steps at $T = 45°\text{C}$ for the $P_3$ and $T_3$ strands respectively, and $1.1 \times 10^{11}$ steps at $T = 60°\text{C}$ for the $P_4$ and $T_4$ strands. Defining melting temperatures of hairpins is complicated because strands may exist in multiple stable structures. We were interested in the point where the strands did not have significantly stable intra-strand base pairs, so we counted all states with at least one intra-strand base pair as 'bound' and states with no intra-strand base pairs as 'unbound', then calculated the yields from Eq. S11. The yield curves for P and T strands are obtained by single histogram reweighting and are shown in Fig. S3, and in Fig. 2 in the main text for just the P strands.

In addition to the melting curves, we also determined free-energy landscapes at $T = 20°\text{C}$ from VMMC simulations with umbrella sampling for the $P_0$, $P_3$, and $P_4$. For $P_0$ strands we used an order parameter that kept track of any intra-strand base pair. For the $P_3$ and $P_4$ strands, we used a two-dimensional order parameter where the two coordinates describe (1) the intended base pairs according to Gao *et al.* [14] ('correct' base pairs) of the structures, and (2) all possible other intra-strand base pairs (total base pairs minus correct base pairs). For a typical strand there was ∼80 possible different intra-strand base pairs. The free energies for the strands were calculated from cumulative distributions from 10 parallel runs with $5 \times 10^{10}$, $5.5 \times 10^{10}$, and $2.5 \times 10^{10}$ steps for $P_0$, $P_3$ and $P_4$, respectively. The results for the free energies and yields of the single strands are shown in Fig. 3 in the main article and in Fig. S3, respectively, and are discussed in Section SIV A 1.

### 2. *Duplex Thermodynamics*

We first computed the melting temperatures of the three duplex systems. For structures consisting of two molecules care must be taken in extrapolating from a simulation of two strands to a bulk solution with many more strands, because fluctuations in local concentrations play an important role. If $\Phi$ is the ratio of bound to unbound states in a simulation of two molecules, the yield of a non-self-complementary duplex in a bulk solution (with the same average concentration of reactants) is given in Ref. 15 as

$$f_{\text{bulk}}^{\text{dim}} = \left(1+\frac{1}{2\Phi}\right) - \sqrt{\left(1+\frac{1}{2\Phi}\right)^2 - 1}. \tag{S12}$$

The melting temperature occurs when $f_{\text{bulk}}^{\text{dim}} = 1/2$, which corresponds to a simulation yield of $\phi = 2$. To compare simulations of single duplexes with experimental data, as in Table SII for the melting temperatures of the 3 systems, $\Phi$ was measured in simulations and then scaled to the experimental concentration ($\Phi$ is proportional to the concentration, so scales from a concentration $c_1$ to another concentration $c_2$ by the factor $c_2/c_1$). The bulk yield was then calculated by using Eq. S12. Note that this approximation only works if the systems are essentially ideal – the accuracy of this approximation has been previously established for oxDNA under similar conditions [15, 16]. We ran 10 VMMC simulations with umbrella sampling for each system using an order parameter that measured the number of inter-strand bonds between the two strands. The simulations for the three systems were carried out at $T = 77.5°\text{C}$, which is near the melting temperature of each system. For $P_0T_0$, $P_3T_3$, and $P_4T_4$, each of 10 simulations ran for $1.6 \times 10^{10}$ steps, $2.9 \times 10^{10}$ steps, and $1.8 \times 10^{10}$ steps, respectively. The results for the duplex yields are shown in Fig. S4 and discussed in Section SIV A 2.

Next, the computations of the relative free energies of the $P_0T_0$, $P_3T_3$, and $P_4T_4$ systems at $T = 20°\text{C}$ were carried out using VMMC moves along with umbrella sampling with a multi-dimensional order parameter that measures (1) the number of intra-strand base pairs in the P strand, (2) the number of intra-strand base pairs in the T strand, and (3) the number of inter-strand base pairs between P and T strands. In the order parameter, any complementary bond is taken into account. This means we include all secondary structural base pairs in the single strands. We ran 10 simulations for $2.3 \times 10^{11}$ steps for the $P_0T_0$ system and for $1.1 \times 10^{11}$ steps for the $P_3T_3$ system. The results are shown in Fig. 5 in the main article for $P_0T_0$, $P_3T_3$, and $P_4T_4$, and discussed in Section SIV A 2.

The same order parameter used for $P_0T_0$ and $P_3T_3$ was initially used for $P_4T_4$. Ten simulations utilizing umbrella sampling each ran for $1.1 \times 10^{11}$ VMMC steps. These results are shown in Fig. 2(c) in the main text. During the course of the simulations, we noticed a difficulty in sampling the 'pseudoknotted' intermediate states (in which the strands were bound by both tails and loops of the hairpins, illustrated in Fig. S6) that were observed in kinetic simulations; the order parameter was not efficient in driving their formation. We sampled these intermediate states in separate simulations with a distinct multi-dimensional order-parameter depending on (1) only the intended 4-stem hairpins in the P strand and (2) in the T strand, (3) the number of base pairs between the loops of the hairpins, (4) the number of base pairs between the two strands not including the loop-loop base

| $Q$ | Description |
|---|---|
| -2 | $d > 5.1\,\mathrm{nm}$ |
| -1 | $1.7\,\mathrm{nm} < d \leq 5.1\,\mathrm{nm}$ |
| 0 | $1.02\,\mathrm{nm} < d \leq 1.7\,\mathrm{nm}$ |
| 1 | $0.57\,\mathrm{nm} < d \leq 1.02\,\mathrm{nm}$ |
| 2 | $d \leq 0.57\,\mathrm{nm}$ & $x = 0$ |
| 3 | $x \geq 1$ |
| 4 | $y \geq 2$ |
| 5 | $y \geq 6$ |
| 6 | $y \geq 15$ |
| 7 | $y = 25$ |

TABLE SI. The order parameter used in FFS simulations of duplex hybridization in all three systems studied. The parameter $d$ is the minimum distance between any intended base pair on the two strands, $x$ is the number of inter-strand base pairs between the two strands which includes mis-aligned and intended duplex base pairs, while $y$ is only the number of intended duplex base pairs between the two strands. For both $x$ and $y$, a base pair is taken to be present if the hydrogen-bonding energy is less than $-0.596\,\mathrm{kcal\,mol^{-1}}$.

pairs, and (5) the correctly aligned duplex base pairs between the strands. We sampled only those states in which at least one intended hairpin base pair was present in each strand, and also only states where coordinate (4) had at least one base pair formed. These simulations allow an estimate of the free energy of the pseudoknotted state compared to the tail-bound state. We ran a set of 10 simulations using this order parameter for $2 \times 10^{11}$ VMMC steps each. These results are shown in Fig. S5 and are discussed in Section SIV A 2.

## B. Kinetics

### 1. *FFS Simulation Details*

In this section we discuss the implementation of the FFS algorithm, discussed in Section SII. As mentioned in Section SII B 1, we simulated the three duplex systems using molecular dynamics at the experimental temperature of $T = 20°\mathrm{C}$. Additionally, in all kinetics simulations we used a simulation box with a volume of $3.96 \times 10^{-23}\mathrm{m}^3$ which corresponds to a concentration of $42\,\mu\mathrm{M}$ that is 21 times larger than the experimental concentration, as was noted in Section SIII. We also use the same definition of a bonded base pair that was discussed in Section SIII.

### 2. *Order Parameter Used in FFS Simulations*

The order parameter used in simulations is detailed in Table SI. Specifically, we use a combination of distance and bond criteria as outlined in Section SIII. Distance criteria are used to define states $Q = -2 \rightarrow 2$, and bonding criteria for states $Q = 2 \rightarrow 7$. For the $Q = 2, 3$ states, we allowed the bond criteria to track any inter-strand bond between the two strands, which allowed us to monitor the number of non-intended inter-strand base pairs (i.e. mis-aligned base pairs) and also the number of correctly aligned inter-strand base pairs that have formed during the initial association events. The bond criteria for states $Q = 4 - 7$ track only correctly aligned inter-strand base pairs.

### 3. *Initial Equilibration of Single-strand States*

Before implementing FFS, we performed lengthy equilibration simulations to ensure that the single strands were initialized in thermodynamically representative states. Here we describe the procedure used to select these states. In Section SIV A 1 the relative free energies for each single strand were computed using VMMC with umbrella sampling. We performed similar simulations except that both P and T strands were simulated in the same box corresponding to a concentration of $42\,\mu\mathrm{M}$. A 3-dimensional order parameter was used that measures (1) the minimum distance between any pairs of nucleotides that are intended to be base pairs in the final duplex, (2) the number of intra-strand base pairs in the P strand, and (3) the number of intra-strand base pairs in the T strand. The strands were prevented from coming within 5.1 nm of each other, as measured by coordinate (1). We ran 10 simulations for $5 \times 10^9$ VMMC steps and saved configurations every $5 \times 10^6$ VMMC steps, which ensured that any two saved configurations were energetically decorrelated from each other. In total we collected a set containing 10000 configurations. For each state described by the 3-dimensional order parameter there is an umbrella bias $w(Q)$. We randomly selected a configuration from the set and saved it to be used in FFS simulations if $w(min)/w(Q) \leq R$, where $R$ is a random number selected within the range $0 < R \leq 1$ and $w(min)$ was the smallest biasing weight applied in the simulation. This step was repeated until 200 configurations were obtained for each of 5 independent FFS simulations. At the start of each flux generation simulation an initial configuration from the saved set of 200 was selected at random and set to be the starting configuration.

### 4. *Second-order Kinetics Approximation*

An important question is whether or not the approximation of instantaneous reactions (i.e. second-order kinetics) is valid for oxDNA. Such an approximation is

reasonable if the time taken from first interaction to full duplex formation or separation of strands is small compared to the diffusional time scale governing the first contact between strands. We did observe simulations spending significant computational times in states where the partially hybridized strands had formed kissing-hairpins. Theoretically, FFS should account for intermediates states with long lifetimes during flux generation. However, as the formation of such states is rare during flux generation, the sampling is poor. As an alternative to the brute-force approach, we assumed second-order kinetics, reducing the sampling challenge during flux generation, and then checked the accuracy of the assumption from the resultant data.

During flux generation, we therefore restarted (from $Q = -2$) trajectories that reached $Q = 3$, the first state in which a bond is present between strands. Consequently, any time spent in configurations with bonds between strands was not measured in our simulations. Technically our overall FFS protocol measured the flux from $Q = -2$ to $Q = 2$, and the subsequent probability of reaching $Q = Q_{\rm max}$ before returning to $Q = -2$. This approximates the flux from $Q = -2$ to $Q = Q_{\rm max}$ provided that the time spent in the intermediate states is small. In this limit, the measured flux is also proportional to the second-order rate constant.

To justify this assumption, we measured the time taken for shooting trajectories launched from intermediate values of $Q = Q'$ to reach $Q = Q' + 1$ or $Q = -2$. We could therefore determine the typical time taken for a configuration starting in the state $Q'$ to either rearrange and proceed to a full duplex ($Q = Q_{\rm max}$), or to dissociate (taken to be when the system reaches the state $Q = -2$), for comparison with the diffusional time scale of first contact. These results are shown and discussed in Section SIV B 1. We find that the second-order approximation is reasonable at the concentration used in the simulations (and would be even better at the experimental concentration, 21 times lower) and therefore the relative fluxes estimated by our approach are decent predictions for the relative rate constants in oxDNA.

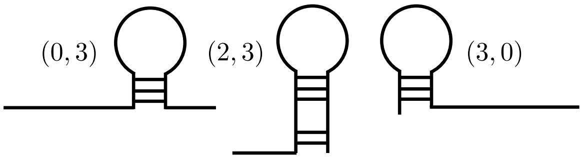


FIG. S2. Schematic representations of the significant hairpin states of the $P_3$ and $T_3$ strands. The $(0, 3)$ state is the hairpin predicted by Nupack to be the most stable, the $(2, 3)$ state is the predicted hairpin with the shorter tail folded in, and the $(3, 0)$ state is a hairpin which appears to be slightly more stable than the predicted hairpin in oxDNA simulations, with the stem at the other end of the strand.

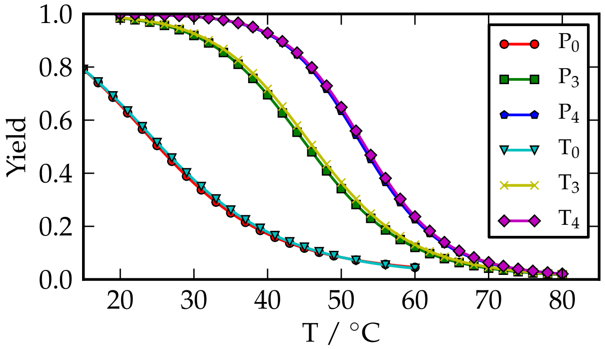


FIG. S3. Yields of all secondary structure of strands as a function of temperature. Error bars on all points are smaller than the symbol size.

## SIV. RESULTS

### A. Thermodynamics

#### 1. *Single-strand Thermodynamics*

Gao *et al.* used the mFold software [17] to design the strands listed in Table 1 in the main article, and assumed the predicted lowest energy structures to be the only important structures in their investigation. mFold uses the nearest-neighbor thermodynamic model developed by Santa Lucia [4] to analyze secondary structure. However, this model cannot yet incorporate more complex structures like pseudoknots or multiple internal loops. It cannot take into account forces that may result from the three-dimensional structure, which can be important in some cases [18].

mFold predicts that at $T = 20°$C the $P_0$ $T_0$ strands, while designed to minimize their secondary structure, had multiple possible structures with free energies close to zero relative to the hairpin-free case, showing the difficulty of eliminating hairpins completely from long strands. From the simulations, we also found that the $P_0$ and $T_0$ strands were not dominated by any particular hairpin, but did frequently have some limited secondary structure. These transient base pairs should have a limited effect on hybridizationas they can melt easily.

To determine the prevalence of stable secondary structure at room temperature, we calculated the yields of the three P strands, which were plotted in Fig. 2 in the main article. Comparable results for the T strands are shown in Fig. S3, which show almost no difference between P and T strands.

OxDNA and mFold disagree slightly about the relative stabilities of similar hairpins within the $P_3T_3$ system. However, these subtleties are likely to be relatively unimportant, as both predict that hairpins are much more stable in $P_3T_3$ than $P_0T_0$, and also much more stable in $P_4T_4$ than in $P_3T_3$. Further, the main hairpin stem is always predicted to be of 3 and 4 base pairs in $P_3T_3$ and $P_4T_4$ respectively.

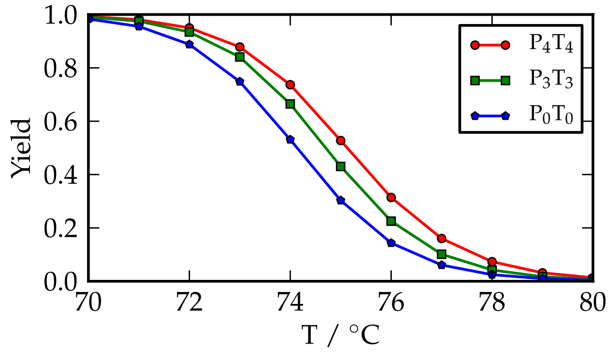


FIG. S4. Yields of all inter-strand structure of the three systems as a function of temperature. Error bars on all points are smaller than the symbol size used.

| Duplex | Gao *et. al* [14] | Santa Lucia [4] | oxDNA |
|---|---|---|---|
| $P_0T_0$ | 76.2 | 76.9 | 74.1 |
| $P_3T_3$ | 77.4 | 77.4 | 74.6 |
| $P_4T_4$ | 78.0 | 77.6 | 75.1 |

TABLE SII. Melting temperatures of P/T duplexes in Celsius at [ssDNA] = 2 µM, as measured experimentally in Ref. 14, calculated with the SantaLucia model [4], and simulated with oxDNA.

### 2. *Duplex Thermodynamics*

Yield curves for bulk solutions in the region of the melting temperature are plotted in Fig. S4 for the three duplex systems, and the melting temperatures are listed in Table SII alongside the experimental values as measured by UV absorbance spectroscopy by Gao *et al.* [14], and the predicted values that were calculated by the Santa Lucia model [4]. All three methods are in agreement as to the order of the melting temperatures, although oxDNA appears to underestimate the true value by around 3°C, corresponding to an error of <1%. This is unlikely to be of significance. What is important for this investigation is that all duplexes melt at temperatures well above room temperature, the order of stability is reproduced, and the differences between the curves is small.

We also computed separately the free energies of the intermediate states in the $P_4T_4$ system relative to the state (5,8), which is shown in Fig. S5, using an order parameter designed to allow the sampling of the pseudoknotted intermediate with inter-strand base pairs between stem and loop. An example of a pseudoknotted intermediate containing 15 inter-strand base pairs and 8 intra-strand base pairs is shown in Fig. S6. The plot in the main text clearly shows a metastable intermediate at (5,8), corresponding to two hairpins bound by their tails. This is also visible in Fig. S5, as is a second local minimum at (13,8) corresponding to the pseudoknotted state. This plot indicates that the pseudoknotted state is less stable than the tail-only state. Kinetic results in Section SIV B show that interchange between the two minima is reasonably fast.

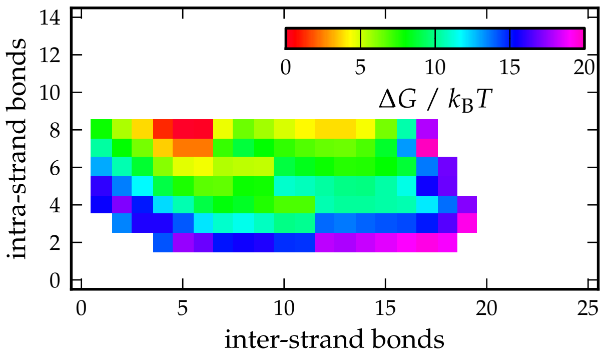


FIG. S5. Free-energy profile of the metastable states in the $P_4T_4$ system. The free energy in both figures is plotted as a function inter- and intra-strand base pairs, where each free energy was measured relative to the state (5,8).

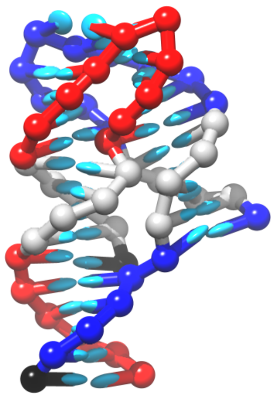

FIG. S6. Example configuration of a metastable kissing complex, found from the FFS simulations of $P_4T_4$. The black nucleotides indicate the 3′ end of the strand, while the gray nucleotides denote intact 4-stem hairpins. In total there are 15 correctly aligned base pairs, 5 connect the tails of the hairpins while 10 base pairs connect the loops of the hairpins.

## B. Kinetics

The hybridization rate constants, $k_+$, the melting rate constants, $k_-$, and the equilibrium constants $K_{eq}$, for each system are listed in Table 2 in the main article. The melting rates, $k_-$, were not computed using FFS, but rather by using Eq. 1 in the main article combined with our calculations of $k_+$ and $\exp(\Delta G^0/k_BT)$ from the free-energy calculations in Section SIV A 2. The cumulative statistics of the FFS simulations for $P_0T_0$, $P_3T_3$, and $P_4T_4$ systems are presented in Tables SIII, SIV and SV, respectively.

| $P_0T_0$ | | | |
|---|---|---|---|
| $\lambda$ | Crossings | Total time | Flux |
| $\lambda_{-1}^{0}$ | 50212 | $1.09 \times 10^{-3}$s | $4.33 \times 10^{7} \pm 2.26 \times 10^{5}\text{s}^{-1}$ |
| $\lambda$ | Success | Attempts | Fractional success |
| $\lambda_0^1$ | 25000 | 78436 | $0.319 \pm 0.002$ |
| $\lambda_1^2$ | 24900 | 116474 | $0.214 \pm 0.004$ |
| $\lambda_2^3$ | 25000 | 489155 | $0.051 \pm 0.002$ |
| $\lambda_3^4$ | 5000 | 36617 | $0.139 \pm 0.010$ |
| $\lambda_4^5$ | 5000 | 7856 | $0.644 \pm 0.036$ |
| $\lambda_5^6$ | 5000 | 5001 | $0.999 \pm 0.0001$ |
| $\lambda_6^7$ | 500 | 500 | $1.000 \pm 0.000$ |

TABLE SIII. Results of FFS for the hybridization of $P_0$ and $T_0$ strands. The flux was measured for the crossing of $\lambda_{-1}^{0}$ and probabilities of reaching $\lambda_{Q-1}^{Q}$ from $\lambda_{Q-2}^{Q-1}$.

| $P_3T_3$ | | | |
|---|---|---|---|
| $\lambda$ | Crossings | Total time | Flux |
| $\lambda_{-1}^{0}$ | 50227 | $1.35 \times 10^{-3}$s | $3.68 \times 10^{7} \pm 2.07 \times 10^{5}\text{s}^{-1}$ |
| $\lambda$ | Success | Attempts | Fractional success |
| $\lambda_0^1$ | 25000 | 88757 | $0.282 \pm 0.001$ |
| $\lambda_1^2$ | 25000 | 125327 | $0.200 \pm 0.003$ |
| $\lambda_2^3$ | 25000 | 638923 | $0.039 \pm 0.002$ |
| $\lambda_3^4$ | 5000 | 26560 | $0.199 \pm 0.020$ |
| $\lambda_4^5$ | 5000 | 12454 | $0.410 \pm 0.031$ |
| $\lambda_5^6$ | 4980 | 5152 | $0.967 \pm 0.006$ |
| $\lambda_6^7$ | 500 | 500 | $1.000 \pm 0.000$ |

TABLE SIV. Results of FFS for the hybridization of $P_3$ and $T_3$ strands. The flux was measured for the crossing of $\lambda_{-1}^{0}$ and probabilities of reaching $\lambda_{Q-1}^{Q}$ from $\lambda_{Q-2}^{Q-1}$.

### 1. *Considerations of the Kinetic Intermediate States*

The formation of the $P_4T_4$ duplex is suggested by Gao *et al.* to have two different kinetic regimes which cannot be fitted to a simple two-state model. They propose a 'fast' regime where the tails of the hairpins bond (or perhaps their loops kiss); and a 'slow' regime where the hairpin stems are displaced by inter-strand base pairs as the strands zip up. The fast regime has a rate constant smaller by a factor of 6, and the slow regime has a rate constant smaller by a factor of 25, than the $P_0T_0$ duplex. In order to see non-second-order behavior, the intermediate state needs to be long-lived so that *both* dissociation and completion of the reaction are slow relative to the association rate.

We find the rearranging time for metastable states to proceed to a full duplex for the majority of simulations to be typically less than $\sim 1 \times 10^{-7}$ seconds for $P_0T_0$ (Fig. S7(a)), and $\sim 1 \times 10^{-5}$ seconds for $P_3T_3$ and $P_4T_4$ (Figs. S7(b) and (c)), while the longest rearrangement times that lead to dissociation events took less than $\sim 1 \times 10^{-5}$ for $P_3T_3$ (Fig. S8(b)) and $\sim 4 \times 10^{-4}$ seconds for $P_4T_4$ (Fig. S8(b)). Comparing the longest rearrangement and disassociation times with the diffusion time, we find for all duplex systems studied that the most long-lived metastable states (in $P_3T_3$ and $P_4T_4$ systems) have a lifetime slightly smaller than the rate at which they are produced. Thus reactions are second order to a reasonable approximation in oxDNA at concentrations of 42 µM (justifying our simulation procedure). Further, our model, consistent with Nupack, suggests that regardless of completion rate, the 6-base pair toeholds (i.e. the tails of the intended 4-stem hairpins) are not stable enough to give rise to two kinetic regimes at the experimental

| $P_4T_4$ | | | |
|---|---|---|---|
| $\lambda$ | Crossings | Total time | Flux |
| $\lambda_{-1}^0$ | 50227 | $1.46 \times 10^{-3}$s | $3.49 \times 10^7 \pm 7.27 \times 10^5 s^{-1}$ |
| $\lambda$ | Success | Attempts | Fractional success |
| $\lambda_0^1$ | 25000 | 77246 | $0.324 \pm 0.003$ |
| $\lambda_1^2$ | 25000 | 113764 | $0.220 \pm 0.002$ |
| $\lambda_2^3$ | 25000 | 856983 | $0.030 \pm 0.002$ |
| $\lambda_3^4$ | 5000 | 23011 | $0.242 \pm 0.037$ |
| $\lambda_4^5$ | 5000 | 16251 | $0.336 \pm 0.050$ |
| $\lambda_5^6$ | 4720 | 21786 | $0.232 \pm 0.025$ |
| $\lambda_6^7$ | 5066 | 5869 | $0.864 \pm 0.014$ |

TABLE SV. Results of FFS for the hybridization of $P_4$ and $T_4$ strands. The flux was measured for the crossing of $\lambda_{-1}^0$ and probabilities of reaching $\lambda_{Q-1}^Q$ from $\lambda_{Q-2}^{Q-1}$.

concentration used. The strands just fall off too quickly, which is unsurprising given the known physics of DNA. We find that the kissing hairpin loops are even less stable, as is the pseudoknotted configuration formed when both the loops and tails bind.

Our simulations do not support the claim of two different kinetic regimes for the formation of the $P_4T_4$ duplex. Therefore, non-second-order behavior in Gao's experiment, if real, must be due to some unknown aspect of DNA thermodynamics that is not incorporated into the oxDNA model.

### 2. *Additional Results for Hybridization Pathways*

In the top panel of Figs. S9 and S10 we plot the frequency that a given base in the P strand is involved in base pairing for configurations that have crossed interfaces $\lambda_{Q-1}^Q$, for $Q = 3, \ldots, 6$, respectively. In the bottom panel of Figs. S9 and S10 we plot the probability that the base pairs between the strands led to a fully-bound duplex state, given that a configuration crossed the $\lambda_{Q-1}^Q$ interface, for $Q = 3, \ldots, 6$, respectively.


[1] Ouldridge, T. E., Louis, A. A., and Doye, J. P. K. (2011) Structural, mechanical and thermodynamic properties of a coarse-grained model of DNA. *J. Chem. Phys.* **134**, 085101.

[2] Ouldridge, T. E. Coarse-grained modelling of DNA and DNA nanotechnology. PhD thesis University of Oxford (2011 [Published as a book by Springer, Heidelberg, 2012]).

[3] Šulc, P., Romano, F., Ouldridge, T. E., Rovigatti, L., Doye, J. P. K., and Louis, A. A. (2012) Sequence-dependent thermodynamics of a coarse-grained DNA model. *J. Chem. Phys.* **137**, 135101.

[4] SantaLucia, Jr., J. (1998) A unified view of polymer, dumbbell, and oligonucleotide DNA nearest-neighbor thermodynamics. *Proc. Natl. Acad. Sci. U.S.A* **17(95(4))**, 1460–5.

[5] Frenkel, D. and Smit, B. (2001) Understanding Molecular Simulation, Academic Press Inc. London UK, .

[6] Whitelam, S., Feng, E. H., Hagan, M. F., and Geissler, P. L. (2009) The role of collective motion in examples of coarsening and self-assembly. *Soft Matter* **5**, 1251–1262.

[7] Torrie, G. M. and Valleau, J. P. (1977) Nonphysical sampling distributions in monte carlo free-energy estimation: Umbrella sampling. *J. Comp. Phys.* **23(2)**, 187–199.

[8] Ferrenberg, A. M. and Swendsen, R. H. (1988) New monte carlo technique for studying phase transitions. *Phys. Rev. Lett.* **61**, 2635–2638.

[9] Russo, J., Tartaglia, P., and Sciortino, F. (2009) Reversible gels of patchy particles: Role of the valence. *J. Chem. Phys.* **131(1)**, 014504.

[10] Verlet, L. (1967) Computer "experiments" on classical fluids. I. Thermodynamical properties of Lennard-Jones molecules. *Phys. Rev.* **159(1)**, 98.

[11] Lapham, J., Rife, J. P., Moore, P. B., and Crothers, D. M. (1997) Measurement of diffusion constants for nucleic acids by NMR. *J. Biomol. NMR* **10**, 252–262.

[12] Murtola, T., Bunkwer, A., Vattulainen, I., and Deserno, M. (2009) Multiscale modeling of emergent materials: biological and soft matter. *Phys. Chem. Chem. Phys.* **11**, 1869–1892.

[13] Ouldridge, T. E., Šulc, P., Romano, F., Doye, J. P. K., and Louis, A. A. (2013) DNA hybridization kinetics: zippering, internal displacement and sequence dependence. *Nucl. Acids Res.* **41(19)**, 8886–8895.

[14] Gao, Y., Wolf, L. K., and Georgiadis, R. M. (2006) Secondary structure effects on DNA hybridization kinetics: a solution versus surface comparison. *Nucl. Acids Res.* **34**, 3370–3377.

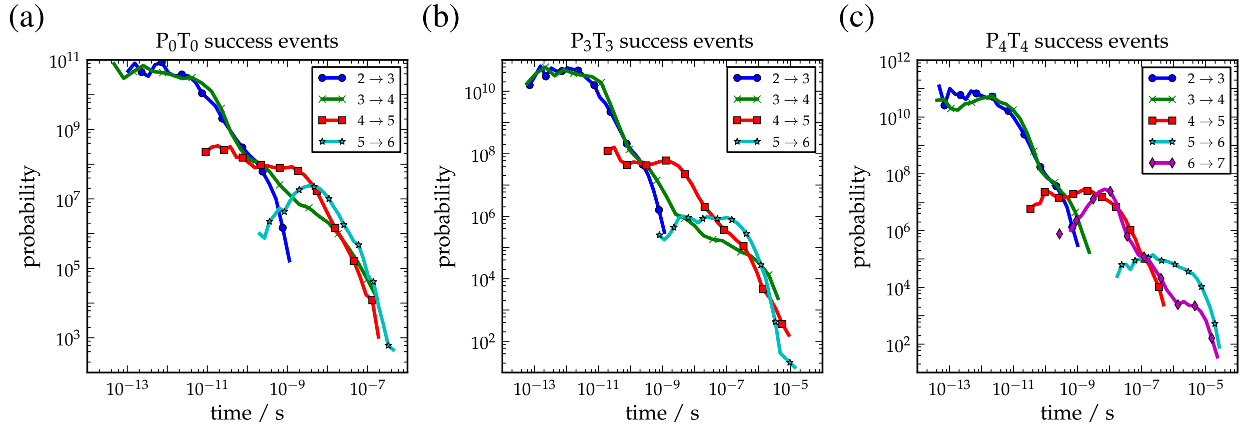


FIG. S7. Histograms of the computed rearrangement times for configurations have started from $\lambda_{Q-2}^{Q-1}$ and crossing $\lambda_{Q-1}^{Q}$ are plotted for (a) $P_0T_0$, (b) $P_3T_3$, and (c) $P_4T_4$. In the legend in each figure, the labels indicate the crossing of a particular interface $\lambda_{Q-1}^{Q}$. For example, $2 \rightarrow 3$ refers to the configuration having crossed interface $\lambda_2^3$ that started from $\lambda_1^2$. The quantity plotted on the $y$-axis is actually a probability density. Note that the times for configurations that crossed $\lambda_6^7$ coming from $\lambda_5^6$ are not plotted as they were found to be neglible.

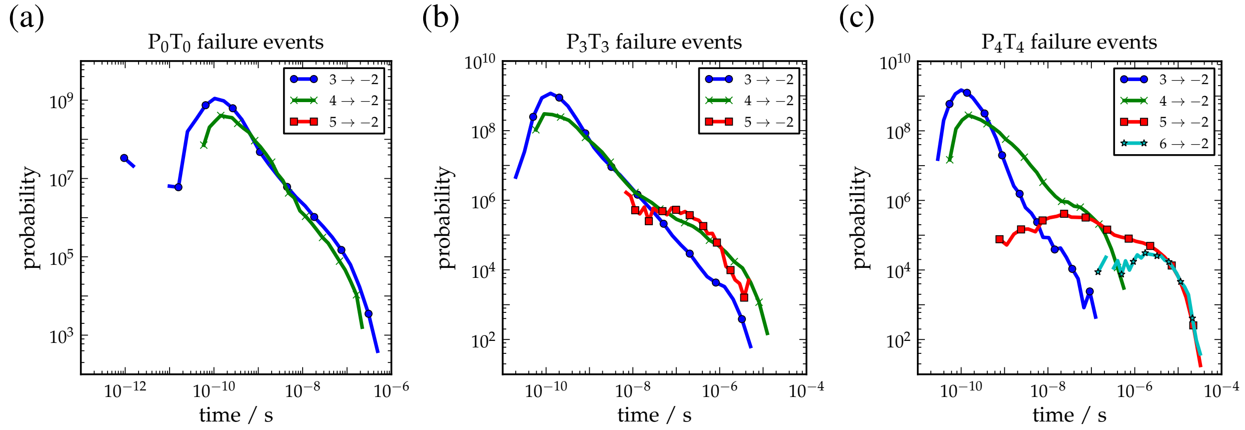


FIG. S8. Histograms of the computed rearrangement times for configurations starting from $\lambda_{Q-1}^{Q}$ and crossing $\lambda_{-2}^{-1}$ before crossing $\lambda_{Q}^{Q+1}$ are plotted for (a) $P_0T_0$, (b) $P_3T_3$, and (c) $P_4T_4$. Similar to Fig. S7, the labels in each legend indicate a configuration having crossed a particular interface $\lambda_{Q-1}^{Q}$. For example, $6 \rightarrow -2$ refers to a configuration having intially crossing $\lambda_5^6$, but crossed $\lambda_{-2}^{-1}$ before crossing $\lambda_6^7$. As in Fig. S7, the quantity plotted on the $y$-axis is actually a probability density. Note that the times for configurations that crossed $\lambda_{-2}^{-1}$ coming from $\lambda_5^6$ are not plotted as they were found to be neglible.


[15] Ouldridge, T. E., Louis, A. A., and Doye, J. P. K. (2010) Extracting bulk properties of self-assembling systems from small simulations. *J. Phys.: Condens. Matter* **22**, 104102.

[16] Ouldridge, T. E. (2012) Inferring bulk self-assembly properties from simulations of small systems with multiple constituent species and small systems in the grand canonical ensemble. *J. Chem. Phys.* **137**, 144105.

[17] Dimitrov, R. A. and Zuker, M. (2004) Prediction of hybridization and melting for double-stranded nucleic acids. *Biophys. J.* **87**, 215–226.

[18] Zhang, D. Y. and Winfree, E. (2009) Control of dna strand displacement kinetics using toehold exchange. *J. Am. Chem. Soc.* **131(47)**, 17303–17314.

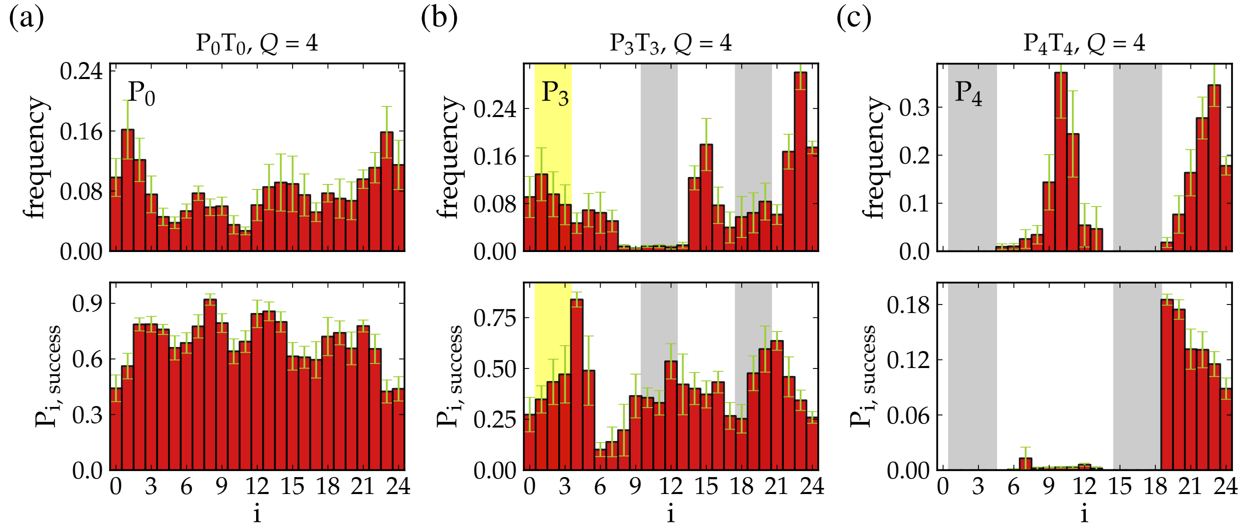


FIG. S9. The frequencies of attachment locations for configurations that crossed $\lambda_3^4$ (labeled state $Q = 4$ in the plots) as a function of base pair index on the P strand are plotted in the top panel in (a) for $P_0T_0$, in (b) for $P_3T_3$, and in (c) for $P_4T_4$. The probability that said base pairs lead to a duplex are plotted in the bottom panels in (a) for $P_0T_0$, in (b) for $P_3T_3$, and in (c) for $P_4T_4$. Similar to Fig. 7, the yellow region indicates a the location of a non-intended 3-base pair hairpin, which pairs with bases at locations 10-12. The grayed out regions for $P_3$ indicate the intended 3-base pair hairpin stem, while the grayed out regions for $P_4$ indicate the 4-base pair hairpin stem. Sites 5-14 are within the loop of the hairpin, and sites 19-24 are a dangling tail.

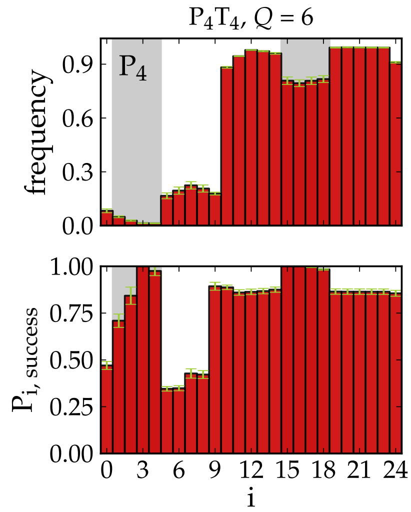


FIG. S10. For $P_4T_4$, the frequencies of attachment locations for configurations that crossed $\lambda_5^6$ (labeled state $Q = 6$ in the plots), as a function of base pair index on the P strand, are plotted in the top panel, and the probability that said base pairs lead to a duplex are plotted in the bottom panel. The grayed out regions for $P_4$ indicate the 4-base pair hairpin stem. Sites 5-14 are within the loop of the hairpin, and sites 19-24 are a dangling tail.
